# Screening for functional transcriptional and splicing regulatory variants with GenIE

**DOI:** 10.1093/nar/gkaa960

**Published:** 2020-11-05

**Authors:** Sarah E Cooper, Jeremy Schwartzentruber, Erica Bello, Eve L Coomber, Andrew R Bassett

**Affiliations:** Wellcome Sanger Institute, Wellcome Genome Campus, Hinxton, Cambridgeshire CB10 1SA, UK; OpenTargets, Wellcome Genome Campus, Hinxton, Cambridgeshire CB10 1SA, UK; Wellcome Sanger Institute, Wellcome Genome Campus, Hinxton, Cambridgeshire CB10 1SA, UK; OpenTargets, Wellcome Genome Campus, Hinxton, Cambridgeshire CB10 1SA, UK; European Molecular Biology Laboratory, European Bioinformatics Institute (EMBL-EBI), Wellcome Genome Campus, Hinxton, Cambridgeshire CB10 1SD, UK; Wellcome Sanger Institute, Wellcome Genome Campus, Hinxton, Cambridgeshire CB10 1SA, UK; OpenTargets, Wellcome Genome Campus, Hinxton, Cambridgeshire CB10 1SA, UK; Wellcome Sanger Institute, Wellcome Genome Campus, Hinxton, Cambridgeshire CB10 1SA, UK; Wellcome Sanger Institute, Wellcome Genome Campus, Hinxton, Cambridgeshire CB10 1SA, UK; OpenTargets, Wellcome Genome Campus, Hinxton, Cambridgeshire CB10 1SA, UK

## Abstract

Genome-wide association studies (GWAS) have identified numerous genetic loci underlying human diseases, but a fundamental challenge remains to accurately identify the underlying causal genes and variants. Here, we describe an arrayed CRISPR screening method, Genome engineering-based Interrogation of Enhancers (GenIE), which assesses the effects of defined alleles on transcription or splicing when introduced in their endogenous genomic locations. We use this sensitive assay to validate the activity of transcriptional enhancers and splice regulatory elements in human induced pluripotent stem cells (hiPSCs), and develop a software package (rgenie) to analyse the data. We screen the 99% credible set of Alzheimer's disease (AD) GWAS variants identified at the clusterin (*CLU*) locus to identify a subset of likely causal variants, and employ GenIE to understand the impact of specific mutations on splicing efficiency. We thus establish GenIE as an efficient tool to rapidly screen for the role of transcribed variants on gene expression.

## INTRODUCTION

Human genetics analysis such as genome-wide association studies (GWAS) and the rise of population scale biobanks are revealing a growing list of genetic loci associated with disease, with >177 000 associations in the GWAS catalog (1). However, due to correlations between genetic variants, known as linkage disequilibrium, the underlying genes and regulatory elements involved are often difficult to ascertain. In most cases, the genetic variants implicated reside within the non-coding genome, and presumably act to regulate gene expression. Statistical colocalisation with expression quantitative trait loci (eQTL) can indicate potential target genes; however, in many cases no colocalised eQTLs are identified (2), while in others multiple genes are implicated (3). Similarly, overlap with epigenomic annotations such as chromatin accessibility, modification or folding can narrow down the list of putative variants. However, none of these methods directly demonstrate causality of a specific variant. Massively parallel reporter assays allow high-throughput assessment of enhancer variants, but are not performed in the endogenous genomic context, and therefore do not recapitulate all of the regulatory features of the native gene (4). Genome engineering approaches in model cell systems circumvent many of these issues and allow the identification of the true causal variants (5). However, the generation and study of isogenic pairs of cell lines is time consuming and there is significant variability during clonal isolation and differentiation that confounds analysis (6), especially of common variants that often have small effect sizes. Thus, there is a pressing need for sensitive and reliable methods to screen for the functionality of large numbers of non-coding variants in their native context.

Here, we develop an arrayed CRISPR screening system, GenIE, that addresses these limitations, and allows investigation of the effect of specific genetic variants and small deletions on gene expression in an endogenous context. We demonstrate that GenIE can assay intronic transcriptional enhancers and splicing regulatory elements in hiPSCs, apply it to screen variants involved in Alzheimer's disease (AD) at the clusterin (*CLU*) locus, and perform saturation editing across a splice site to quantify the effects of point mutations on splicing.

## MATERIALS AND METHODS

### Ethics approval and consent

iPSC lines were generated as part of the HipSci project (KOLF2, Cambridgeshire 1 NRES REC Reference 09/H0304/77) or (corrected A1ATD iPSCs, Hertfordshire NRES REC Reference 08/H0311/201) and work on these is covered under HMDMC 14/013.

### GenIE method

The rgenie package for R implements the statistical analysis and visualisations reported herein, beginning from aligned amplicon sequence data. It is available at https://github.com/jeremy37/rgenie. Details of regions targeted with GenIE are in Supplementary Table S2. All guide sequences, HDR oligo sequences, and primer sequences used herein are detailed in Supplementary Tables S3–S5. A summary of statistical results for each targeted SNP is in Supplementary Table S6. Details of all GenIE replicates are in Supplementary Table S7. A single replicate was excluded (out of 696), for MUL1 (rs6700034 locus) due to low read count.

### Experimental design

The GenIE method requires the SNP of interest to be within the transcribed unit, i.e., coding, UTR or intronic and that this gene is transcribed (TPM >1) in the cells to be assayed. We chose the guide with a cut site closest to the SNP of interest which had either a NGG PAM (for SpCas9) or a NGA PAM (for VRQR SpCas9). Off-target cutting of the guides was checked using WGE (https://www.sanger.ac.uk/htgt/wge/) and guides with multiple off-target cutting with a mismatch of 1 or 2 nucleotides were not used if there was another suitable guide near (within ∼10 bp of) the SNP. Off-target effects are less critical in a GenIE experiment as only the gene of interest is assayed, although guides which cut the genome multiple times can be toxic and therefore were avoided if possible. Full-length chemically synthesised and modified sgRNAs (Synthego) were used. An HDR oligo (100 bp Ultramer, IDT) containing the SNP of interest was designed and the sense of the oligo that was used was dependent on the position of the cut site of the guide relative to the SNP (7) (if cut site was to the right of the SNP a sense oligo used, and vice versa). For SNPs that were heterozygous in KOLF2-C1 hiPSCs, we designed a second-site mutation in between the SNP and the cut site of the guide, so that we could distinguish the edited allele from the non-edited allele. If the guide did not cover the SNP, or the SNP was the N within the PAM, then we designed a second site mutation to avoid recutting of the guide after HDR. For experiments that required a second-site mutation, we designed an HDR oligo that included the second-site mutation and SNP mutation together, and another HDR oligo that included the second-site mutation alone. We mixed these oligos together (70:30 molar ratio respectively) when carrying out the editing to generate appropriate alleles to assay any effect of the second-site mutation.

Primers were designed to amplify the region surrounding the SNP of interest and contained adaptor sequences for the addition of barcodes for Miseq (Supplementary Table S3) (8). The amplicons were less than 295 bp (ideally around 200 bp) to allow sequencing using a 150 paired-end Miseq run. For the analysis of splicing events using GenIE we designed primers within the neighbouring exons to amplify from mature RNA. All primers were unique in BLAT searches.

A primer for gene-specific reverse transcription was also designed for each SNP of interest in the opposite direction to the direction of the transcription of the gene and about 30–50 bp outside of the amplicon primers. Using such a primer in the reverse transcription reaction increased sensitivity and allowed better amplification of nascent RNA.

### hiPSC cell culture

Human KOLF2-C1 (HIPSCI, www.hipsci.org) or corrected A1ATD iPSCs (9) were grown in feeder-free conditions in TeSR-E8 medium (Stemcell Technologies) on Synthemax (Corning) (final amount 2 μg/cm^2^) and routinely passaged 1:10 every 5 days using Gentle Cell Dissociation Reagent (Stemcell Technologies).

### Arrayed CRISPR–Cas9 editing

hiPSCs were edited by nucleofection of RNP complex (containing full-length guide RNA and SpCas9), along with an ssODN repair template (10). Briefly, SpCas9 and the VRQR variant (11) were expressed and purified from *E.coli* using a His-tag. The purified protein was diluted to 4 μg/μl in storage buffer (10 mM Tris–HCl pH 7.4, 300 mM NaCl, 0.1 mM EDTA, 1 mM DTT). Full-length guides (Synthego) were resuspended in TE (200 μM) and diluted to 45 μM in duplex buffer (IDT). Diluted SpCas9 (1 ul, 4 μg, 24.2 pmol) was mixed with diluted guide (1 μl, 45 pmol) and left at RT for 10–20 min for RNP complexes to form. The ssODN repair template was added (1μl, 100 pmol) to the RNP complex just before the nucleofection. Cells were washed once with PBS, and a single-cell suspension was harvested using accutase (8 min at 37°C). Cells were washed in TeSR-E8 plus ROCK inhibitor, counted and resuspended in P3 buffer. Screening of up to 16 SNPs at once was possible using small nucleofection cuvettes (V4XP-3032 Lonza) with final amounts per nucleofection 2 × 10^5^ cells, 20 μl P3 buffer, 1 μl (4 μg) SpCas9, 1 μl (45 pmoles) sgRNA and 1 μl (100 μM) HDR oligo. Cells were electroporated using 4D-Nucleofector on program CA137. After nucleofection cells were plated onto a 6-well dish coated with Synthemax (5 μg/cm^2^) with TeSR-E8 supplemented with Rock inhibitor. After 24 h, the media was exchanged for TeSR-E8 and after 5–7 days cells were split to 10 cm dishes.

Editing of a smaller number of SNPs was carried out using large cuvettes (V4XP-3024 Lonza), with the same conditions except the final amounts per nucleofection were 1 × 10^6^ cells, 100 μl P3 buffer, 5 μl (20 μg) SpCas9, 5 μl (225 pmol) sgRNA and 5 μl (500 pmol) HDR oligo. After nucleofection the cells were plated onto 10 cm dishes.

Cells were grown to ∼80% confluence in a 10 cm dish (5–7 days) and then harvested by accutase. Cell pellets were washed once in PBS before flash freezing on dry ice and stored at –80°C. Routinely six identical cell pellets, each containing 2 × 10^6^ cells were harvested from a single 10 cm dish.

### Genomic DNA isolation

Genomic DNA was prepared using the MagAttract HMW Kit (Qiagen, 67563). The frozen cell pellets were resuspended in 180 μl Buffer ATL and 20 μl proteinase K, transferred to a 2 ml eppendorf and lysed for 1 h at 65°C at 900 rpm. gDNA was then extracted from the lysate following the manufacturer's protocol and eluted from the beads in 100 μl DNase-free water. PCR was carried out using PowerUp SYBR Green Master Mix (Applied Biosystems) with 5 μl gDNA (250–500 ng) template and 0.4 μM final concentration forward and reverse primers in a 50 μl reaction. The PCR reaction mixture was split into four tubes (12.5 μl) for amplification to avoid founder biases and then repooled ready for barcoding PCR. Typically four PCRs were carried out from a gDNA preparation.

**Table:** 

95°C 10 min	1 cycle
95°C 15 s	
57°C 15 s	30 cycles
62°C 30 s	
62°C 5 min	1 cycle

### RNA isolation and reverse transcription

RNA was extracted using the Direct-zol RNA Miniprep kit (Zymo R2071) following the manufacturer's protocol. It was important to use a trizol-based extraction method to allow the successful purification of nascent nuclear RNA. We used 300 μl TRI-Reagent to resuspend the frozen cell pellets straight from dry ice. We carried out the optional in-column DNase digest and RNA was eluted from the column in 50 μl DNAse/RNAse-free water. We then performed a further DNase treatment of the RNA using TURBO DNA-free kit (Thermo-Fisher) as the manufacturer's protocol. We made cDNA from 1 μg RNA using Superscript IV (Thermo-Fisher) according to the manufacturer's protocol. Importantly we used a gene-specific reverse transcription (RT) primer (final concentration: 0.1 μM) to prime the reverse transcription as this increased sensitivity when amplifying from low abundance, nascent RNA. Typically two reactions of cDNA synthesis (20 μl each) and a control lacking the RT enzyme were carried out using these conditions.

**Table:** 

50°C	10 min
55°C	10 min
60°C	10 min
80°C	10 min

PCR was carried out using PowerUp SYBR Green Master Mix (Applied Biosystems) with 5 μl cDNA template and 0.4 μM final concentration forward and reverse primers in a 50 μl reaction. The PCR reaction mixture was split into 4 tubes (12.5 μl) for amplification to avoid founder biases and then repooled. Typically, 8 PCRs were carried out from one RNA preparation.

**Table:** 

95°C 10 min	1 cycle
95°C 15 s	
57°C 15 s	30 cycles
62°C 30 s	
62°C 5 min	1 cycle

The PCR products were analysed on a 2% agarose gel, stained with ethidium bromide, alongside the minus RT controls before the barcoding PCRs were carried out.

### CCDC6 splice site mutagenesis

To perform the saturation mutagenesis experiment, HDR templates were designed for each base alteration, and were mixed together in equimolar amounts before nucleofection. Two nuclefections were carried out to ensure high enough levels of HDR (>1%) for each event, with nucleofection 1 altering 17 bases and nucleofection two altering 16 bases. A common HDR template was also added to both nucleofections to allow for a comparison between them. The final amounts per nucleofection were 1 × 10^6^ cells, 100 μl P3 buffer, 5 μl (20 μg) SpCas9, 5 μl (225 pmol) CCDC6 sgRNA and 5 μl (500 pmol) of the mixture of HDR oligos.

### Sequencing

In order to add the Miseq indices (8) we performed a second PCR using 1 μl PCR1 (from gDNA and cDNA), PowerUp SYBR Green Master Mix (Applied Biosystems), and 0.4 μM final concentration forward and reverse primers in a 25 μl reaction.

### ATAC-seq

We carried out ATAC-seq on KOLF2-C1 iPSCs (three samples, cultured as above) and also on iPSC-derived cortical neurons.

Differentiation of iPSC-derived cortical neurons was carried out as described in Shi *et al.* (12). Briefly iPS cells were induced to form a monolayer of NPCs by addition of dual SMAD inhibition and by WNT signalling inhibition for 12 days. After 16 days NPCs were dissociated with accutase and plated a low density on laminin to form neurons. ATAC samples were taken at Day 35 from three independent differentiations.

ATAC-seq was performed as described in Kumasaka *et al.* (13). Briefly, a single cell suspension of iPSCs or iPSC-derived cortical neurons was made using accutase, and nuclei were extracted before undergoing tagmentation using the Illumina Nextera kit. Each ATAC sample was made from 100 000 cells. After PCR amplification and size selection the ATAC libraries were sequenced on Hiseq 4000 with an average of ∼100 million reads per sample.

We downloaded 9 microglial ATAC-seq datasets based on the study by Gosselin *et al.* (14). We aligned ATAC-seq reads for the three hiPSC samples, three neuronal samples, and nine microglial samples to GRCh38 with bwa 0.7.15. We prepared bigWig files from alignments by using bedtools genomecov, followed by bedGraphToBigWig. For display purposes (Figure 2) we combined all samples within each cell type.

### hiPSC QTL fine-mapping

To identify candidate causal variants in hiPSCs, we used summary statistics for gene eQTLs and sQTLs from a large study of hiPSCs (15), and for each of these QTL types filtered to retain genes with at least one tested SNP having association *P* < 1 × 10^−5^. We used the Wakefield method (16) to determine SNP approximate bayes factors from summary statistics, and then applied WTCCC-style fine-mapping (17) assuming a single causal variant per QTL to determine SNP posterior probabilities. For gene-level eQTLs this identified >470 SNPs with greater than 99% probability each of causally affecting gene expression. We examined the top few candidates to identify transcribed SNPs within ATAC-seq peaks in hiPSCs, and selected SNPs in *MUL1* (rs6700034) and *ABHD4* (rs8011143) for GenIE editing. For sQTLs, we additionally annotated SNPs with their score from SpliceAI (18), and from among the many SNPs with both high causal probability and high SpliceAI score we selected SNPs in *TAF1C* (rs4150126) and *SDF4* (rs60252802) for editing. To generate the plots in Figures 2A and 3A, we obtained anonymised sample genotypes and normalised gene expression or splice junction usage values from the i2QTL consortium data (15), and plotted values by genotype using the ggbeeswarm R package.

### Selection of *CLU* SNPs and GenIE experiments

SNPs in *CLU* associated with Alzheimer's disease were identified previously, as described in Schwartzentruber *et al.* (19). The locus likely contains two causal variants, one in *PTK2B* and one in *CLU*. Mean fine-mapping probabilities for SNPs in the *CLU* region are given in Supplementary Table S1.

The 11 CLU SNPs were processed in three separate GenIE batches, which had differing numbers of cDNA and gDNA replicates: batch A was done as 3 cDNA or gDNA preparations followed by three PCR replicates for each extraction, for a total of 18 replicates; batch B was done identically, except that nine PCR replicates were done from the first gDNA preparation, and three from each of the other two; batch C was done with two cDNA preparations followed by four PCR replicates each, and one gDNA preparation followed by four PCR replicates. To make all SNPs comparable, we downsampled the experiments with more replicates (batches A and B) to match batch C by selecting eight cDNA and four gDNA replicates for each SNP, which were balanced across the cDNA and gDNA preparations. Supplementary Table S7 provides details of the replicates used. In all cases, the downsampled results were comparable (very similar effect size estimates) to those obtained using all performed replicates.

### Read alignment and quality control

Since all amplicons were smaller than 300 bp, we first merged the overlapping 150-bp paired-end reads using FLASH v1.2.11 (20) to improve alignment of Cas9-induced deletions. As input to FLASH we specified a minimum overlap of 10 bp, fragment size as the amplicon size, fragment standard deviation of 20, and maximum mismatch density of 10%, and used the –allow-outies parameter. A mean of 94% of reads could be successfully merged, with standard deviation of 8%. We aligned merged reads to a human reference containing the sequences of all amplicons using bwa mem v0.7.17 (21), with lenient parameters to allow aligning Cas9-induced deletions (-O 24,48 -E 1 -A 4 -B 16 -T 70 -k 19 -w 200 -d 600 -L 20 -U 40).

For each replicate (cDNA or gDNA) at each locus, the rgenie software extracts reads mapping to the targeted region from the aligned BAM file. Different analyses were done to quantify the effects of targeted SNP changes (HDR events) vs. deletions. For HDR analysis, no reads were discarded, and we used ‘grep’ to identify reads with either the HDR or WT allele, requiring a match of 6 nt on each site of the altered site. For deletion analyses, reads were discarded if they had any insertions, if they did not span the site of SNP change, if they aligned to <30 bp of the amplicon, or if they had a mismatch fraction >5%. The read cigar string was used, along with read start coordinates, to identify whether a read matched the HDR or WT allele at the SNP site (with no requirement to match in a specific window around this apart from the above filters), or to identify positions in the amplicon where the read had a deletion. Deletion reads were never considered as HDR or WT. Reads were considered to have a Cas9-induced deletion if they had any deletion that spanned the window ±20 bp from the cut site. Deletion reads were represented internally as a ‘unique deletion profile’ (UDP), such that reads with identical deletions but one or more mismatches were considered to be the same allele.

### Statistical analysis

To identify gene expression differences for an allele X (either HDR or deletion allele), we first determine for each replicate the ratio of the read count of X to that of the WT allele:

(1) $$\begin{equation*} r = \frac{{read\ count\ X}}{{read\ count\ WT}} \end{equation*}$$

This normalisation ensures that we can accurately estimate the fold-change effect of allele X (relative to WT) even when either X or WT represents a large or small proportion of total reads. (Note that while in principle the WT read count could be zero, in practice we would not analyze an experiment where this is the case, and we recommend only considering experiments / replicates where ≥5–10% of reads are WT.) We thus have ratios {*r^C^_1_, r^C^_2_, …, r^C^_N_*} for *N* cDNA replicates, and ratios {*r^G^_1_, r^G^_2_, …, r^G^_M_*} for *M* gDNA replicates. We use these ratios, rather than direct counts, because following PCR and deep sequencing there will likely be duplicate reads from individual DNA molecules. Note also that because cDNA and gDNA are extracted separately, followed by independent PCRs, there is no pairing between individual cDNA and gDNA replicates. Therefore, we separately compute the mean ratio in cDNA, $\overline {{r^C}} = \frac{1}{N}\sum\nolimits_{i = 1}^N {{r^C}_i}$, and the mean ratio in gDNA, $\overline {{r^G}} = \frac{1}{M}\sum\nolimits_{i = 1}^M {{r^G}_i}$. If allele X alters the expression level of the gene in which it appears (relative to WT), then the ratios will differ between cDNA and gDNA, i.e. $\overline {{r^C}}$ ≠ $\overline {{r^G}}$. Since the variance of gDNA replicates tends to be lower, we use a two-tailed unequal variances (Welch's) *t*-test to test for a difference in this ratio.

We define the effect size β as:

(2) $$\begin{equation*}\beta = \frac{{\ \overline {{r^C}} \ }}{{\ \overline {{r^G}} \ }}\end{equation*}$$

This effect size represents the fold change in expression of an allele, relative to the WT allele, normalizing for the prevalence of the allele within the DNA of the cell population. Note that rather than testing for a difference in means, $\overline {{r^C}}$ ≠ $\overline {{r^G}}$, a nearly equivalent test would be β≠1. In practice, the test for a difference in means is more straightforward, and for significance testing, we report *P* values from this test. However, for visualization we report effect size estimates β, and determine 95% confidence intervals as follows. We denote the standard deviation of the cDNA ratios *r^C^* as ${\sigma _C} = \sqrt {\frac{1}{{(N - 1)}}\sum\nolimits_{i = 1}^N {{{({r^C}_i - \overline {{r^C}} )}^2}} }$, with standard error of the mean ($\overline {{r^C}}$) as ${\rm{S}}{{\rm{E}}_{\rm{C}}} = {\sigma _C}/\sqrt N$. The corresponding values for gDNA are defined similarly: ${\sigma _G} = \sqrt {\frac{1}{{(M - 1)}}\sum\nolimits_{i = 1}^M {{{({r^G}_i - \overline {{r^G}} )}^2}} }$, and ${\rm{S}}{{\rm{E}}_{\rm{G}}} = {\sigma _G}/\sqrt M$. To determine a confidence interval for β, we first determine the uncertainty (standard deviation σ_β_) of the estimate β using the propagation of uncertainty formula for a ratio β = *A*/*B*:

(3) $$\begin{equation*}{\sigma _\beta }^2\ \approx \ {\beta ^2}\left[ {{{\left( {\frac{{{\sigma _A}}}{A}} \right)}^2}\ + \ {{\left( {\frac{{{\sigma _B}}}{B}} \right)}^2}\ - \ 2\frac{{{\sigma _{AB}}}}{{AB}}} \right]\end{equation*}$$

where *A* = $\overline {{r^C}}$, σ_A_ is the standard deviation of *A*, *B* = $\overline {{r^G}}$, and σ_*B*_ is the standard deviation of *B*. Note that because *A* and *B* in this ratio are the estimated means, the uncertainty in these means is their standard error. That is, ${\sigma _{\rm{A}}} = {\rm{S}}{{\rm{E}}_{\rm{C}}} = {\sigma _{\rm{C}}}/\sqrt N$, and ${\sigma _{\rm{B}}} = {\rm{S}}{{\rm{E}}_{\rm{G}}} = {\sigma _{\rm{G}}}/\sqrt M$. We take the covariance σ_*AB*_ to be zero since the replicates are independent. (This is conservative, since a nonzero value for the covariance would reduce the uncertainty.) We estimate the degrees of freedom for an unequal variances *t*-test using the Welch–Satterthwaite equation:

(4) $$\begin{equation*}\nu \ \approx \ \frac{{{{\left( {\frac{{{\sigma _C}^2}}{{{N^2}}}\ + \ \frac{{{\sigma _G}^2}}{{{M^2}}}} \right)}^2}}}{{\frac{{{\sigma _C}^4}}{{{N^2}\left( {N - 1} \right)}}\ + \ \frac{{{\sigma _G}^4}}{{{M^2}\left( {M - 1} \right)}}\ }}\end{equation*}$$

We then determine the 95% confidence interval of β as:

(5) $$\begin{equation*}95\% {\rm{ CI}} = [{\rm{\beta }} - {{\rm{t}}_{0.975}}*{\sigma _{\rm{\beta }}},{\rm{\beta }} + {{\rm{t}}_{0.975}}*{\sigma _{\rm{\beta }}}].\end{equation*}$$

where *t*_0.975_ is the the 0.975 quantile of the t distribution with degrees of freedom *v*.

### Variance components analysis

To determine the variance attributable to different experimental factors, we performed GenIE at eight intronic SNPs within different genes, using multiple repeats at different stages of the process: one round of Cas9 editing, three repeats each of genomic DNA extraction and RNA extraction/reverse transcription, three replicates of PCR each for gDNA and cDNA, two repeats of barcoding, and two repeats of sequencing (separate Miseq runs), for a total of 576 replicates (Supplementary Figure S4). One region did not edit successfully, and so was excluded (72 total replicates). For each sequenced replicate we determined the fraction of reads representing each unique allele (‘unique deletion profile’, UDP), relative to the total number of reads. We then used the variancePartition R package (22), with all replicates at a given locus, to determine variance components attributable to each factor (cDNA/gDNA extraction, PCR, barcoding, and sequencing) for the fraction of reads for each allele. These are displayed separately for cDNA and gDNA replicates (Supplementary Figure S4).

### Power analysis

To estimate the power of a GenIE experiment to identify effects of specific alleles, we used the presence of multiple different unique deletion alleles present at different fractions. For each allele, we determined its fraction, relative to all reads in the replicate, and computed the mean (*f*) and the coefficient of variation (CV, standard deviation divided by the mean) of this fraction across replicates. In general, the coefficient of variation is higher for alleles with lower abundance. We then fit a curve to predict CV from the mean allele fractions separately for cDNA and gDNA (Supplementary Figure S3a), and found that the following form gave a reasonable fit:

(6) $$\begin{equation*} CV = a + \frac{b}{{\sqrt f }} \end{equation*}$$

Here, *a* and *b* are parameters that are fit for a given experiment. We restricted the alleles considered to those with >0.1% frequency. Using the fit, we can estimate the CV expected in either cDNA or gDNA for a given allele fraction, and consequently, the standard error (SE) of the estimate as CV / $\sqrt N$. We then use the propagation of uncertainty formula for a ratio (Eq. 3) separately to determine the standard error of the cDNA estimate *r^C^* = (*f*_A_ / *f*_WT_), the gDNA estimate *r^G^* = (*f*_A_*/ f*_WT_), and the standard deviation of the effect size β = *r^C^ / r^G^*, at any given allele frequency *f*_A_ and number of cDNA replicates *N* and gDNA replicates *M*. Because the power to detect an effect of a given allele depends on the variability in the WT quantification as well, we used the observed CV of the WT allele (separately in cDNA and gDNA) in all power calculations. The *t* score is then determined as *t* = |β – 1|/σ_β_, and the power is 1 – 2*P(–|*t*|) with degrees of freedom estimated as for the Welch *t*-test (Eq. 4) based on the chosen number of replicates. For power estimates reported in the main text, we computed power separately at each of 13 targeted regions (MUL1, ABHD4, TAF1C and 10 CLU SNPs which excludes CLU_4-rs4236673 since editing failed at that SNP), based on the variability of allelic estimates within those regions, and assumed eight cDNA and four gDNA replicates. These values are in Supplementary Table S8, and we reported the minimum power across the 13 regions.

## RESULTS

### Genome engineering based interrogation of enhancers (GenIE)

**Figure 1.**
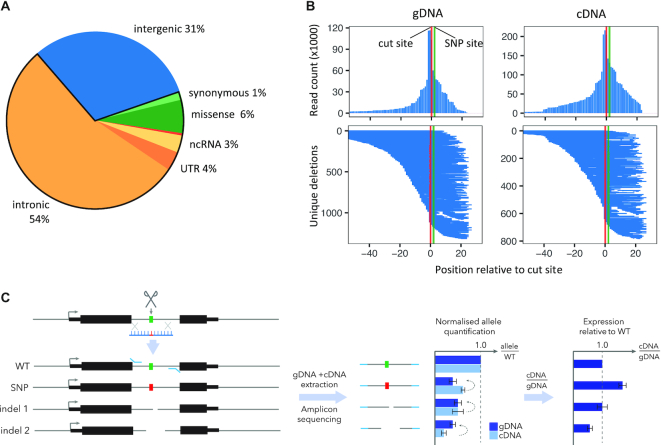
GenIE overview. (**A**) Proportion of GWAS lead SNPs in different genomic regions; 69% fall in transcribed regions (introns/exons/splice sites/UTRs/ncRNAs). (**B**) Deletion profiles from Cas9 editing at *MUL1* intronic SNP rs6700034 in hiPSCs, assayed in genomic DNA (gDNA, left) and complementary DNA (cDNA) generated from RNA (right). (top row) count of sequencing reads having a deletion at each nucleotide position relative to the cut site; (bottom row) profile of each unique Cas9-induced deletion. (**C**) Schematic of GenIE assay. Edited pools of cells contain a mixture of WT, edited point mutation (SNP) and a variety of deletion alleles (indel 1, 2, etc.), expression from each of which can be quantified by amplicon sequencing of cDNA and gDNA extracted from the same population of cells.

Whereas only 6% of GWAS lead SNPs alter protein-coding sequence, nearly 70% of disease-associated variants are within transcribed regions, the majority of which are intronic (Figure 1A) (1). Building on our previous work (23), we developed the GenIE assay to assess the effects of single nucleotide variants residing within intronic regions on either gene expression or splicing using versatile hiPSC-based model systems.

We first deliver Cas9 ribonucleoprotein along with a ∼100 nt ssDNA oligonucleotide homology directed repair (HDR) template into hiPSCs (10). This generates a mixed population of cells containing unedited (WT) alleles, the desired genetic variant and a large number of distinct small insertions and deletions (Figure 1B, C). Next, we extract genomic DNA (gDNA) and RNA from this cell population, and perform multiple replicates of PCR using an amplicon spanning the edited site in both gDNA and cDNA, followed by high-throughput sequencing. Gene expression of each allele (or collections of alleles) is calculated as the ratio of sequencing reads in cDNA relative to gDNA. Within each replicate, the expression of a given allele is normalised to the unedited (WT) reads to identify any change in gene expression relative to WT. By adjusting the PCR primers used for the assay, it can be adapted to measure gene expression or specific splicing events.

**Figure 2.**
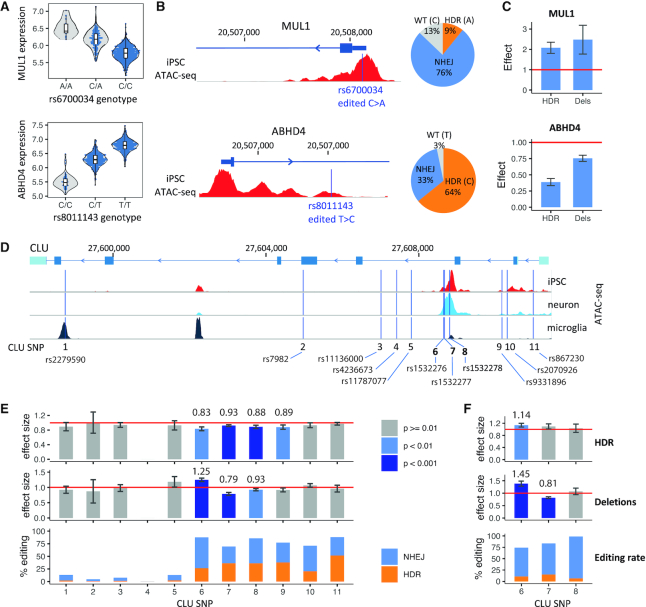
GenIE identifies effects of intronic enhancer elements. (**A**) Violin plots of eQTLs for *MUL1* and *ABHD4* in hiPSCs. (**B**) (left) Genomic position of targeted SNPs within *MUL1* and *ABHD4* enhancer elements; (right) pie chart showing the corresponding editing rates. (**C**) Barplots of GenIE-measured expression of alternative alleles (HDR-introduced allele or deletion alleles) in hiPSCs, relative to WT allele. (**D**) *CLU* gene region, showing ATAC-seq profiles from hiPSCs, hiPSC-derived neurons, and primary microglia, with positions of 11 targeted SNPs indicated. (**E**) GenIE-measured expression of HDR-introduced alleles or deletions, relative to the WT allele in hiPSCs. Effect sizes (fold change) of significant results are shown above each bar. (**F**) GenIE expression for *CLU* SNPs 6–8, relative to WT, with editing in an hiPSC line homozygous for the opposite haplotype; for each SNP, the edit was in the opposite direction to the edit in panel (c). All error bars represent 95% confidence intervals.

We optimised GenIE for lowly-expressed intronic sequences (Supplementary note, Supplementary Figures S1, S2, Methods) and applied it across 13 intronic SNPs. Based on the variability of allele quantification in gDNA and cDNA, we estimate that we can detect a 1.2-fold change in expression for alleles present at ∼1% frequency with >70% power when using 12 PCR replicates (8 cDNA and 4 gDNA) (Supplementary Figures S2–S4, Methods and Supplementary Table S8). We also developed an R package (rgenie, https://github.com/Jeremy37/rgenie) that automates analysis of experiments, including statistical assessment of HDR and small deletions, quality control, and analysis of the deletion repertoire (Figure 2, Supplementary Figures S5–S9, S12, S14).

### Intronic enhancers

We performed fine-mapping of hiPSC eQTLs (15) to identify candidate variants with a high probability of causally influencing hiPSC gene expression. We applied GenIE to a variant in the 5′ UTR of *MUL1* (rs6700034) and a second variant in the first intron of *ABHD4* (rs8011143), both of which overlapped with accessible chromatin regions defined by ATAC-seq (Figure 2B). For *MUL1*, GenIE estimated elevated expression of the A allele relative to C (2.1×, *P* = 4.5 × 10^−5^, Welch's *t*-test) (Figure 2C), consistent with the eQTL effect (Figure 2A). Interestingly, small deletions spanning rs6700034 showed a similar upregulation of *MUL1* expression (2.4×, Figure 2C, Supplementary Figures S5 and S6), suggesting that this region may normally bind a repressor which is disrupted by either deletions or by the A allele. Indeed slightly longer deletions show a larger upregulation, suggesting that there is an extended binding site (Supplementary Figures S6 and S7). This demonstrates how the analysis of the deletion repertoire generated in a GenIE experiment can provide useful information about the mechanism of action of the variant of interest. We repeated the experiment with a second Cas9 single guide RNA (sgRNA) and obtained a very similar effect (Supplementary Figure S7), showing that the results were independent of the sgRNA identity.

For the *ABHD4* eQTL, the intronic rs8011143-C genotype correlates with decreased *ABHD4* expression in hiPSCs (Figure 2A). As expected, conversion of the T>C genotype gave a decrease in expression in the GenIE experiment (0.4×, *P* = 3.3 × 10^−4^, Welch's *t*-test, Figure 2B). Deletions showed a similar yet smaller decrease in *ABHD4* expression (0.8×, Supplementary Figure S8 and S9).

We next applied GenIE to screen 11 variants at the clusterin (*CLU*) locus that form the 99% confidence set of credibly causal variants identified by fine mapping of an AD GWAS (19). The *CLU* gene has been implicated in AD progression, likely due to an effect on amyloid beta aggregation or clearance (24), and *CLU* knockout is neuroprotective in rodents (25–27) and in hiPSC neurons (28). All 11 variants were located within the transcribed unit (Figure 2D, Supplementary Table S1), and included several within putative enhancers as defined by ATAC-seq, along with a synonymous variant in exon 5. The 11 regions showed a substantial variability in overall editing efficiency and HDR rates (Figure 2E), although there was only one case (#4) where the rates of editing were too low (<1%) to interpret a result. As previously observed (29), high editing rates were often associated with regions of accessible chromatin as identified by ATAC-seq, which makes these gene regulatory elements particularly amenable to GenIE analysis. Four SNPs showed a significant (*P* < 0.01, technical replicates, Welch's t-test) reduction in gene expression (0.8–0.9×), three of which (variants 6, 7, 8) sit within a single ATAC peak in intron 3 that is present in hiPSCs and neurons (Figure 2D, Supplementary Figure S10). This set of three SNPs also showed an effect of small deletions (*P* < 0.01, technical replicates, Welch's *t*-test), with deletions at one of these showing increased expression, the opposite effect direction relative to SNP introduction. To further investigate this result, we performed GenIE on these three SNPs (variants 6, 7 and 8) within the intron 3 ATAC peak using an hiPSC line that was naturally homozygous for the opposite haplotype at this locus. As expected, deletions over variants 6 and 7 showed the same result as before. However in this case there was no effect at variant 8 (Figure 2F). We also observed no significant changes in expression upon introduction of variants 7 and 8 in this haplotype. This may be explained by biological effects such as interactions with other variants within this haplotype, but also may indicate that for small effect sizes identified in a GenIE screen, additional repeats may be necessary to confirm screening results. When effect sizes are small, the power to detect a change in expression is modest (e.g. at most 75% power at 5% expression change, Supplementary Figure S3). Detection of small effects may also be more dependent on subtle biological differences in cell state between experiments, or to technical factors, such as the specific genome edits that occur or the alignment of sequencing reads. Thus, statistical significance, effect size and editing rates should be considered when interpreting the results of a GenIE screen. Nevertheless, and consistent with our previous result, a C>T conversion at variant 6 showed a significant upregulation in expression, opposite in direction to the effect of a T>C conversion in the alternative haplotype (Figure 2F) and highlighting this variant as worthy of further investigation.

Taken together, these data show the effectiveness of GenIE in identifying causal effects of individual SNPs in UTRs and introns on gene expression.

### Splicing analysis

A large number of transcribed variants are predicted to have a role in regulating alternative splicing or alternative polyadenylation (30). We postulated that by judicious positioning of primer pairs either within or outside exons, we could use GenIE to detect changes in splicing by either a gain or loss of the spliced or unspliced isoforms (Supplementary Figure S11).

**Figure 3.**
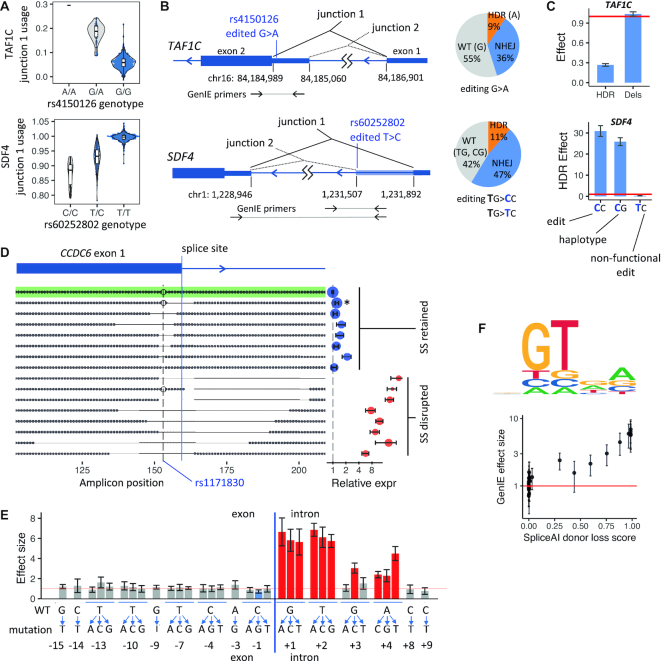
GenIE identifies splicing regulatory elements. (**A**) Violin plots of splicing QTLs for *TAF1C* (usage of junction 1, chr16:84184989–84186901) and *SDF4* (junction 1, chr1:1228946–1231892) in hiPSCs. (**B**) (left) Genomic region showing differential splicing at *TAF1C* and *SDF4* loci; (right) fraction of reads for HDR, deletion (NHEJ) and wild-type (WT) alleles for *TAF1C* and *SDF4*. (**C**) GenIE-measured expression of targeted SNP alleles. (**D**) Deletion profiles of the top 16 alleles by read count from GenIE targeting of rs1171830. Shown on the right is the expression of each allele relative to the WT (reference) SNP allele, coloured by whether the canonical splice site motif is retained (blue) or disrupted (red). (**E**) GenIE-measured expression for dense mutagenesis near the *CCDC6* exon1-intron1 splice site. All error bars represent 95% confidence intervals. (**F**) (top) Sequence logo showing that GenIE recapitulates the consensus splice site motif, with letter size proportional to the inverse of the GenIE effect size when mutated to that nucleotide (relative to the WT/consensus, set to 1); (bottom) Scatter plot showing correlation between GenIE-measured effect size and SpliceAI score for donor splice site loss.

To identify candidate variants likely to affect splicing, we performed fine-mapping of splice quantitative trait loci (sQTLs) in hiPSCs. The rs4150126 variant in the 5′ UTR of *TAF1C* showed a strong sQTL effect, whereby the A allele was associated with more frequent usage of a downstream splice acceptor site (Figure 3A, junction 1). We placed one PCR primer within the alternatively spliced region and the second in the common part of exon 2. Therefore, if the upstream splice site (junction 2) was used, both primers would be within the same exon, but if the downstream splice acceptor (junction 1) was used, one primer would be within intron 1, and thus only detect nascent (pre-spliced) transcripts, resulting in an apparent reduction in expression. GenIE analysis showed that conversion of rs4150126 G>A resulted in a strong reduction in expression (0.3×), whereas small deletions around rs4150126 had no effect (Figure 3B, C, Supplementary Figure S12). These results are consistent with a mechanism whereby the A allele creates a novel splice acceptor site (Figure 3B, junction 1), in which case deletions would not be expected to have any effect. Importantly, it also demonstrates a causal effect of rs4150126 within this haplotype.

As a second example we examined *SDF4*, where rs60252802 (T>C) associates with gain of a splice donor site and an extension of exon 1. To assay this effect, we placed one PCR primer in the extended region of exon 1 and a second in exon 2 (Figure 3B). Thus, extension of exon 1 results in both primers being in the mature transcript, whereas otherwise only one primer would be within the spliced mRNA. As this amplicon would be too large to amplify from genomic DNA, we used an additional nearby primer to assess the frequency of alleles in the genomic DNA (Figure 3B). This design does not allow interpreting the effects of deletions (which will not be seen in the RNA amplicon) but is highly sensitive for detecting a SNP effect on splicing. The hiPSC line we used for GenIE was heterozygous at rs60252802, and consistent with this, we observed the extension of exon 1 in a proportion of RNA-seq reads (Supplementary Figure S13). To assay the effect of rs60252802 independent of haplotype effects, we introduced two HDR templates during Cas9 editing, namely, the two alleles of rs60252802 (C or T, in bold) in conjunction with a common, nearby second-site mutation (C>G). Thus, in one GenIE experiment we could determine the effect of the haplotype (**C**G to **T**G, 25× upregulation), the effect of the SNP and second-site mutation together (**C**C to **T**G, 30x upregulation), and the effect of the second-site mutation alone (**T**C to **T**G, downregulation) with respect to the T haplotype (Figure 3C, Supplementary Figure S13).

We reasoned that we could also apply GenIE in a multiplexed format, whereby we introduce multiple mutations across a defined region in the same pool of cells. We targeted a region of the *CCDC6* gene neighbouring a splice donor site, which includes the synonymous AD risk SNP rs1171830. There was no effect of A>C conversion of the SNP (95% CI 0.95–1.03). However, the apparent expression of deletions removing the splice site (red) was 15-fold higher than those retaining the splice site (blue, Figure 3D, Supplementary Figure S14), likely due to extension of the exon to include the second primer binding site. This demonstrates how we can use the information encoded in the deletion repertoire to (re)discover regulatory elements. We then designed HDR template oligos to mutate every base around the splice site to every other possible base (avoiding amino acid substitutions), a total of 33 single nucleotide changes. We performed GenIE using pools of 16 or 17 variants in each electroporation to ensure HDR rates of >1% per allele. As expected, all variants in the canonical splice donor site (GT) at the beginning of the intron had a strong effect on splicing, along with certain base substitutions of the subsequent two nucleotides (Figure 3E). The effects measured by GenIE recapitulated the splice donor site consensus sequence of GTRA (Figure 3F), and strongly correlated with effect size predictions from SpliceAI (18), a machine learning-based method of splice site prediction (Figure 3F).

## DISCUSSION

Although CRISPR-mediated genome editing and isolation of clonal cell lines provides one means to establish causal effects of noncoding genetic variants, the number of candidate variants overwhelms our ability to produce and characterise clonal cell lines. We have described an arrayed CRISPR screening method, GenIE, that can be used in an unbiased manner to assess the modest effect sizes of common genetic variants on transcription and splicing with high power, and which is scalable to hundreds of SNPs. Importantly, variants are introduced at their endogenous locus and therefore are subject to all of the gene regulatory layers that exist in the natural context. Together, our results demonstrate the effectiveness of GenIE in assaying the effects of genetic variants on transcription and splicing and to define the location and critical sequence motifs of functional elements. While we have used hiPSCs as a convenient model system, GenIE is compatible with differentiation of cells into disease-relevant cell types, and in principle could also be applied to cell types where clonal isolation is difficult, such as primary cells.

Although GenIE can potentially assay the ∼70% of GWAS variants that lie within transcribed regions of the genome, there remains a need for additional methods that assay non-transcribed regulatory elements, or those that exist outside of the gene they regulate. Methods have been developed to understand the effects of gene regulatory elements by repressing their function using catalytically inactive Cas9 fused to transcriptional repression (KRAB) domains (31,32), or by introducing small indels (33). Whilst applicable to non-transcribed regulatory elements, these techniques are restricted by their resolution (∼1 kb for KRAB repressor domains), and their inability to assay the single nucleotide changes identified by GWAS or other human genetics studies, which can give different effects to deletion of the underlying regulatory element. Also, despite extensive optimisation of editing efficiency (10), 49% of sites we tested (19 of 39) gave HDR rates below a usable level. We anticipate that further developments in the genome engineering field such as biasing DNA repair (34), base editing (35,36) or prime editing (37) will improve our ability to perform such screens, and expand the number of variants that can be screened still further.

## DATA AVAILABILITY

The R package (rgenie) developed in this study is available at https://github.com/Jeremy37/rgenie

Raw data is available at Zenodo (https://doi.org/10.5281/zenodo.4044006) and processed data in Supplementary Table S7.

## Supplementary Material

gkaa960_Supplemental_Files
