# Supplementary material for: Screening for functional transcriptional and splicing regulatory variants with GenIE
Source: Nucleic Acids Res. 2020 Nov 5;48(22):e131. doi: 10.1093/nar/gkaa960 (PMC7736817; doi:10.1093/nar/gkaa960)
Supplement: gkaa960_Supplemental_Files [file gkaa960_supplemental_files.zip › GenIE supplement.pdf]

Supplementary Information

Screening for functional transcriptional and splicing regulatory variants with GenIE

Consisting of supplemental notes and supplementary figures 1-14

Supplemental Notes

Optimisation and assessment of the sensitivity of GenIE experiments

We tested the reproducibility of GenIE on a mixture of two hiPSC lines that expressed BFP or GFP from a single copy integration at the *ROSA26* locus. This showed that across varying proportions of the two lines, the proportion of GFP reads in gDNA and cDNA were very well correlated with each other (r^2=0.98, Figure S1). We next applied GenIE to intronic sequences, since transcriptional enhancers frequently reside within intronic regions of the genes that they regulate, with an enrichment within the first intron. Importantly, the level of intronic expression has been shown to correlate better with transcriptional rates than the level of mature transcript(1), making this assay highly amenable to investigating transcriptional regulatory elements. Since introns are expressed at lower levels than exons, we needed to optimise cell lysis and reverse transcription to obtain a reproducible amplification from low frequency events within the complex mixture of alleles. The most important modifications were to use a Trizol-based lysis to extract nascent, unspliced RNAs, and the use of a gene specific reverse transcriptase primer binding adjacent to the intronic sequence to be assayed.

We performed GenIE at 8 intronic SNPs within an intronic region using multiple repeats at different stages of the process, and analysed the variation deriving from each stage (Supplementary Fig. 4, Methods). This showed variability was higher for cDNA than for gDNA, and that noise from PCR amplification contributed the most to variability, whereas RNA reverse transcription, experimental batch, barcoding and sequencing were only minor contributors (Supplementary Fig. 4). This demonstrated that the absolute amount of cDNA inputted into the PCR was the most critical parameter, and we developed our protocol to optimise sensitivity and scalability using the maximal amount of cDNA in each PCR reaction (Supplementary Fig. 2, 4, Methods). We optimised power by increasing the number of cDNA replicates relative to gDNA, to account for increased variability in cDNA replicates. We recommend at least 3 gDNA replicates, and at least twice the number of cDNA replicates as gDNA replicates. We also analysed our power to detect different levels of gene expression change across different loci, which showed that the assay was extremely sensitive, but that this was highly dependent on the frequency of a particular allele within the population (Supplementary Fig. 3). Across multiple sites, we estimate that we are able to detect a 1.2-fold change in expression for alleles present at ~1% frequency with >=70% power when using 12 PCR replicates (8 cDNA and 4 gDNA). The frequency of WT reads within a sample also affected our ability to detect gene expression changes, and in those cases where total editing was >98%, experiments were redone with ⅓ of the amount of Cas9 ribonucleoprotein complex to reduce editing efficiency. For all experiments shown here, we thus limited our analysis to those loci with >1% HDR and >5% WT reads.

Analysis of heterozygous regions

Heterozygosity complicates the GenIE experiment, since we have to classify reads according to whether they are from the A haplotype, B haplotype or edited A haplotype. To achieve this, we design sgRNAs that are specific to one haplotype (i.e. overlap with the SNP of interest), and introduce in the HDR template a second site mutation that is very close to the SNP of

interest, thus marking the edited events (e.g. **C**C, where the bold C is the SNP of interest and the other C is the second site mutation). To control for an effect of the second site mutation by itself, we also include a second HDR template that contains the second site mutation but not the SNP of interest (e.g. **T**C).

Supplementary Figures

1. BFP/GFP cell mixing experiment shows that GenIE is highly reproducible
2. Schematic of experimental design for GenIE screen
3. Power estimates
4. Variance components analysis
5. MUL1 deletion profile, UNS plot
6. QC plots for MUL1
7. MUL1 sgRNA2 deletion profile, UNS plot, QC plots
8. ABHD4 deletion profile, UNS plot
9. QC plots for ABHD4
10. *CLU* supplementary figures
11. Examples of primer positioning for GenIE
12. “Deletion profile plot” and “replicate QC plot” for TAF1C
13. SDF4 splicing genome browser view and replicate QC plot (only HDR/WT ratios for each mutation across repeats) for SDF4
14. “Deletion profile plot” and “replicate QC plot” for CCDC6

Supp Fig 1

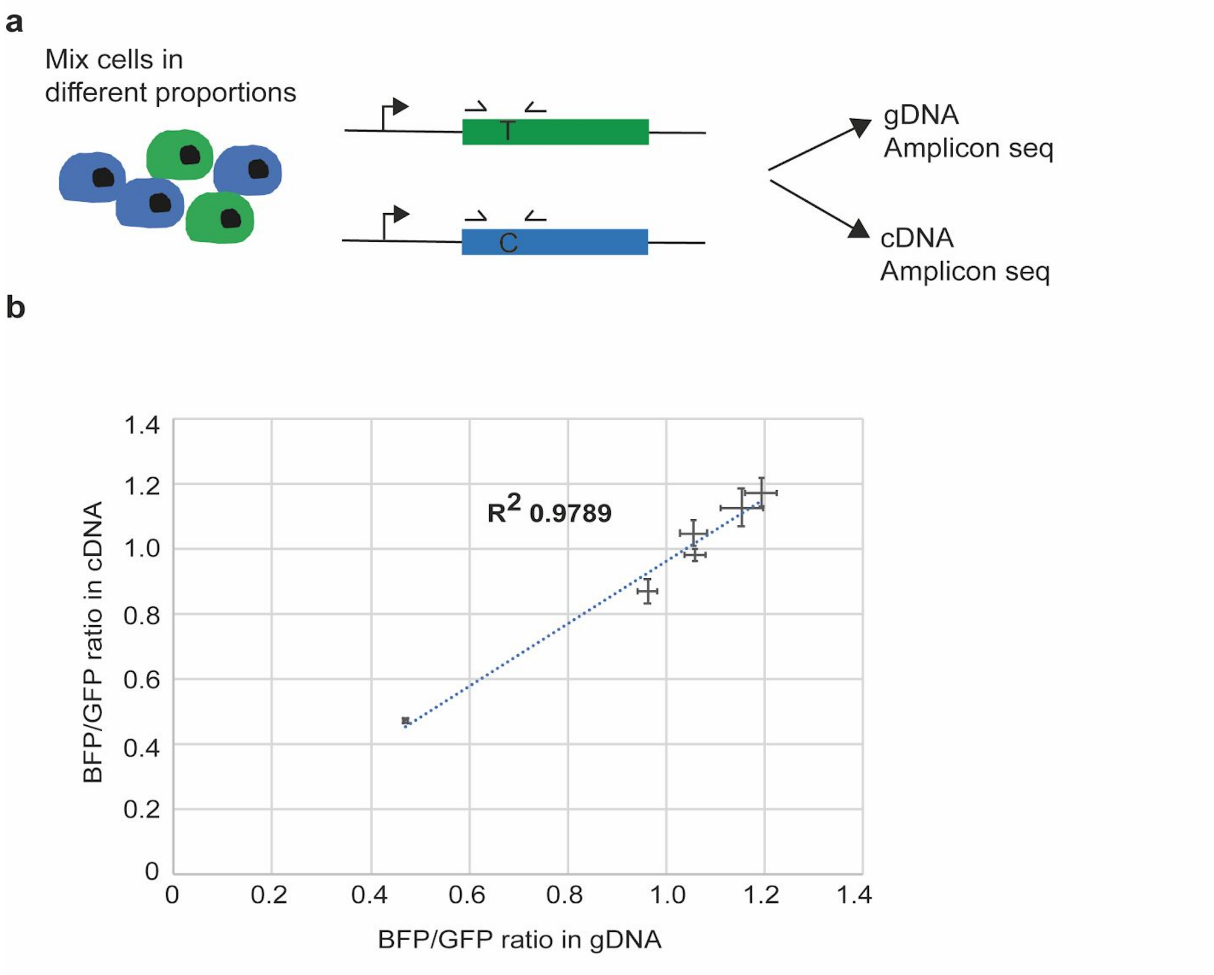

Supplementary Figure 1: BFP/GFP cell mixing experiment shows that GenIE is highly reproducible. a) Two hiPSC lines that express BFP or GFP from a single copy integration at the ROSA26 locus were mixed at a variety of different proportions. BFP and GFP alleles differed by a single base change. gDNA and cDNA were made from each mixed population of cells and amplicon sequencing was performed to quantify the number of BFP and GFP alleles. b) The ratio of BFP/GFP reads in the gDNA was plotted against the BFP/GFP ratio in cDNA, and shows a high correlation between gDNA and cDNA (R^2=0.9789). Error bars show standard deviation, n=4. Dotted line shows linear regression.

Supp Fig 2

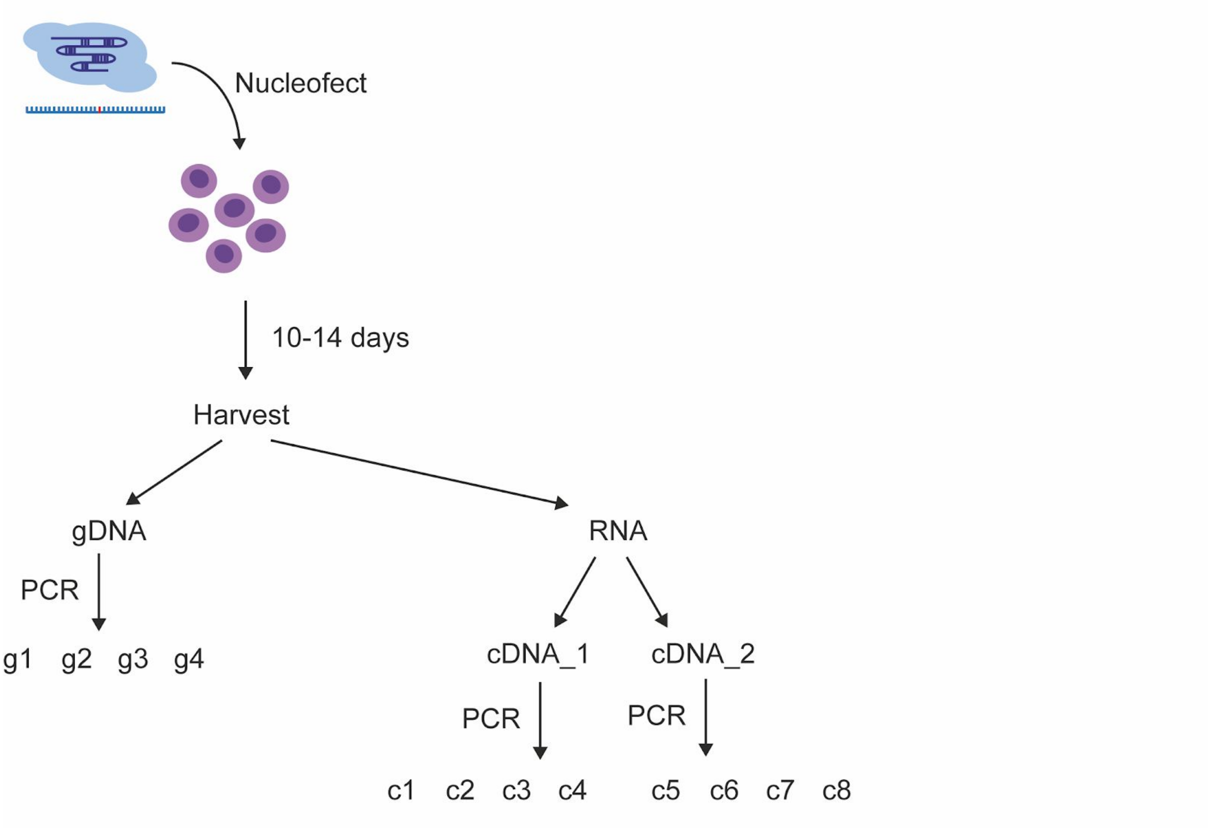

Supplementary Figure 2: Schematic of experimental design for GenIE screen. hiPSCs are nucleofected with RNP (eSpCas9 complexed with full length modified sgRNA) and a ssODN. After 10-14 days in total, cells are harvested with accutase, and pellets are flash frozen. Genomic DNA is extracted and routinely 4 PCR repeats are carried out. RNA is extracted and routinely 2 cDNA preparations (and 1 control lacking reverse transcriptase) are made. From each cDNA preparation, 4 PCR repeats are carried out giving a total of 8 PCR repeats. All PCRs are indexed and sequenced by MiSeq.

Supp Fig 3

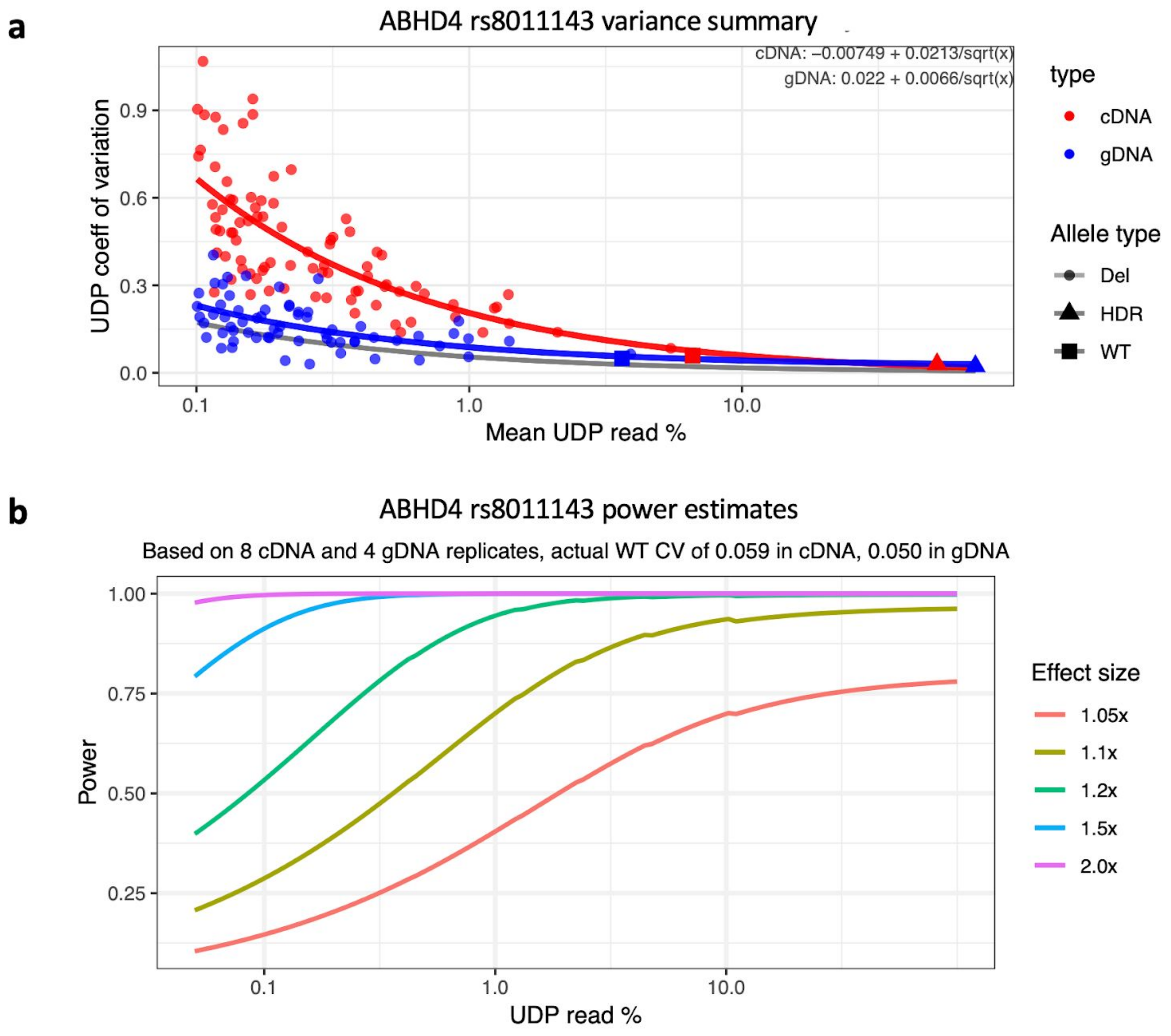

Supplementary Figure 3: Power estimates. The power to detect a causal effect of an allele depends upon the variability in quantification of this allele, and that of the WT allele, across replicates, as well as upon the number of replicates. **(a)** A plot of the coefficient of variation (CV) of measured allele fraction (in the edited pool) across replicates for alleles with at least 0.1% frequency from GenIE editing at the *ABHD4* SNP rs8011143. Separate curves for gDNA and cDNA are fit with the formula a + (b / sqrt(UDP freq)). **(b)** A plot of estimated power at different effect sizes and allele fractions. To estimate the expected power for an allele with a given fraction in the edited population, we use the estimated CV for an allele at that frequency, and the observed CV for the WT allele, and then propagate uncertainty in the ratio $(UDP_{cDNA} / WT_{cDNA}) / (UDP_{gDNA} / WT_{gDNA})$. Slight discontinuities in the power curves are due to changing estimated integer degrees of freedom for the *t* test. See the methods for details.

Supp Fig 4

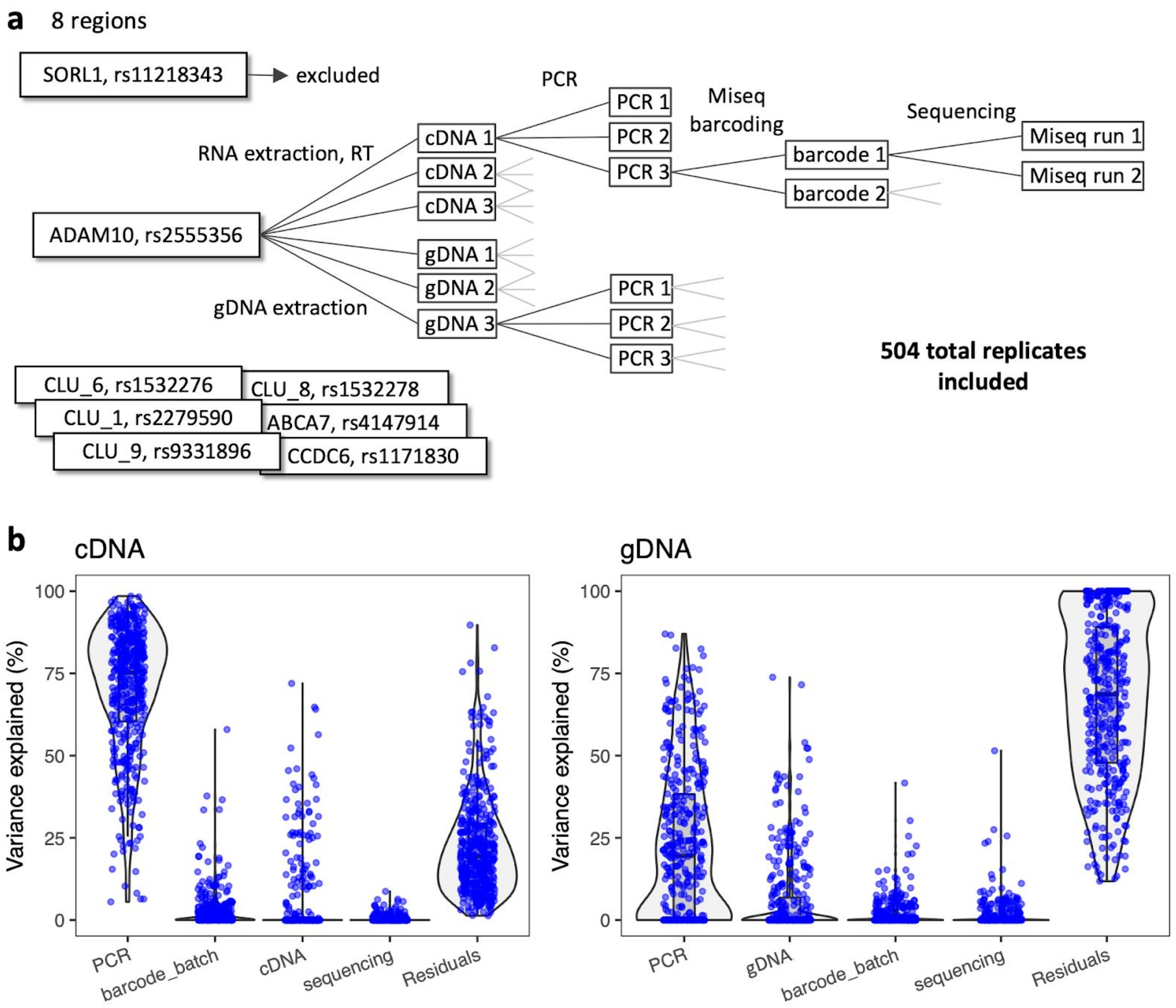

Supplementary Figure 4: Variance components analysis. **(a)** To identify which experimental factors contribute most to variability in GenIE measurements, we performed GenIE at 8 intronic regions. One region was excluded due to poor editing. For each region we did three separate extractions of genomic DNA and 3 RNA extractions / reverse transcriptase reactions; for each of these we did 3 PCRs; for each of these we added 2 distinct barcodes. We put all barcoded PCRs onto two separate MiSeq sequencing runs. **(b)** For each distinct allele, we quantified the fraction of reads for the allele relative to all locus reads, excluding alleles individually representing < 0.1% of reads. We used the variancePartition R package (separately for cDNA and gDNA) to determine the fraction of variance attributable to RNA/DNA extraction, PCR, barcoding, and sequencing. For cDNA, most variation comes from PCR; for gDNA, PCR again contributes the most to variability, but the majority of the variability remains unexplained.

Supp Fig 5

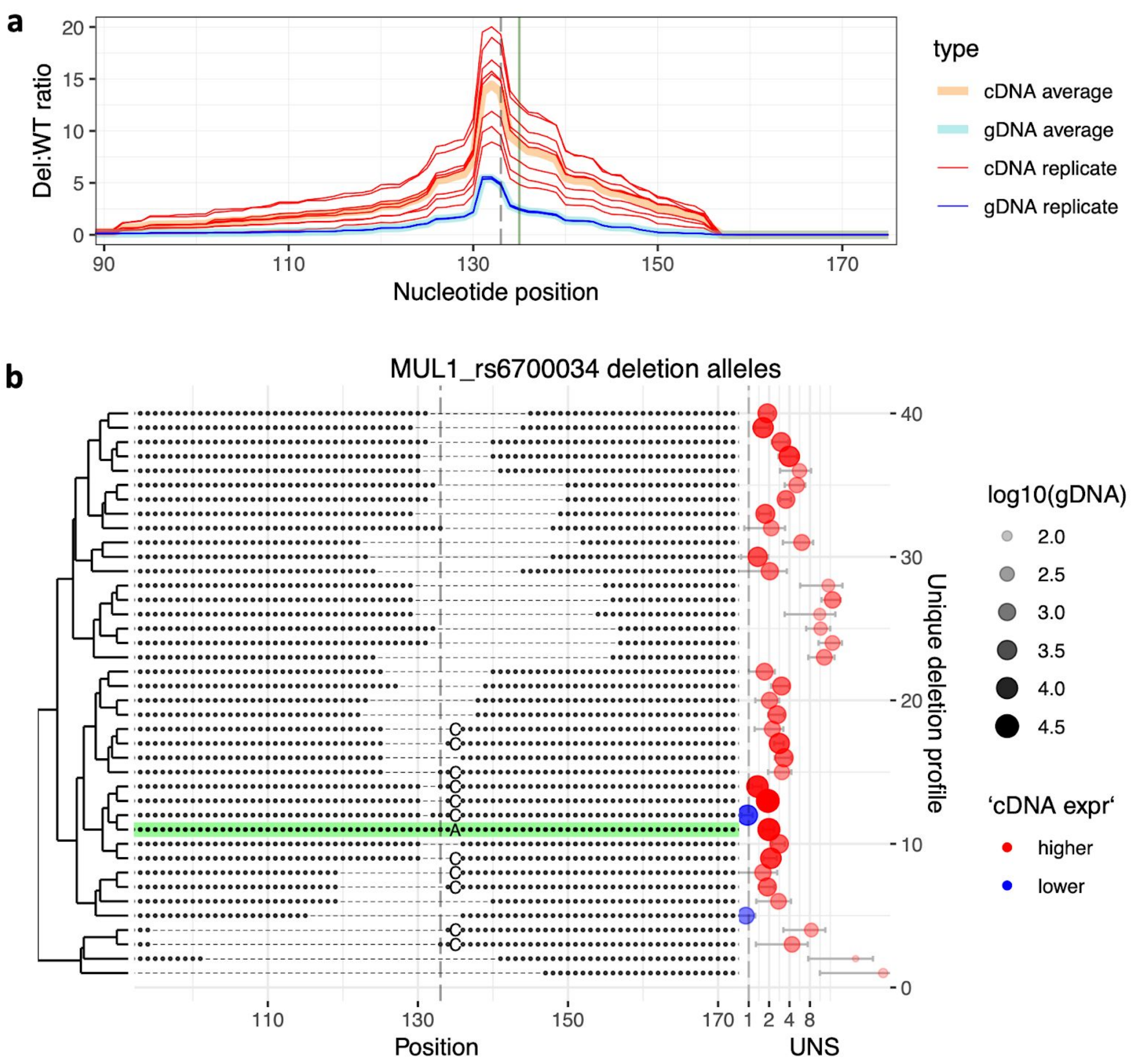

Supplementary Figure 5: *MUL1* GenIE deletions. **(a)** A deletion profile plot shows, for each position in the *MUL1* amplicon (80 bp window centred on rs6700034), the fraction of reads with a deletion covering the position, divided by the total number of WT reads. Reads with deletions are upregulated in cDNA relative to their prevalence in gDNA. **(b)** A UDP-normalized score (UNS) plot shows, for the top 40 alleles, the deletion profile of the allele, as well as its estimated expression relative to WT (UNS score). Alleles are clustered according to their deletion profile, and the HDR allele is highlighted green.

Supp Fig 6

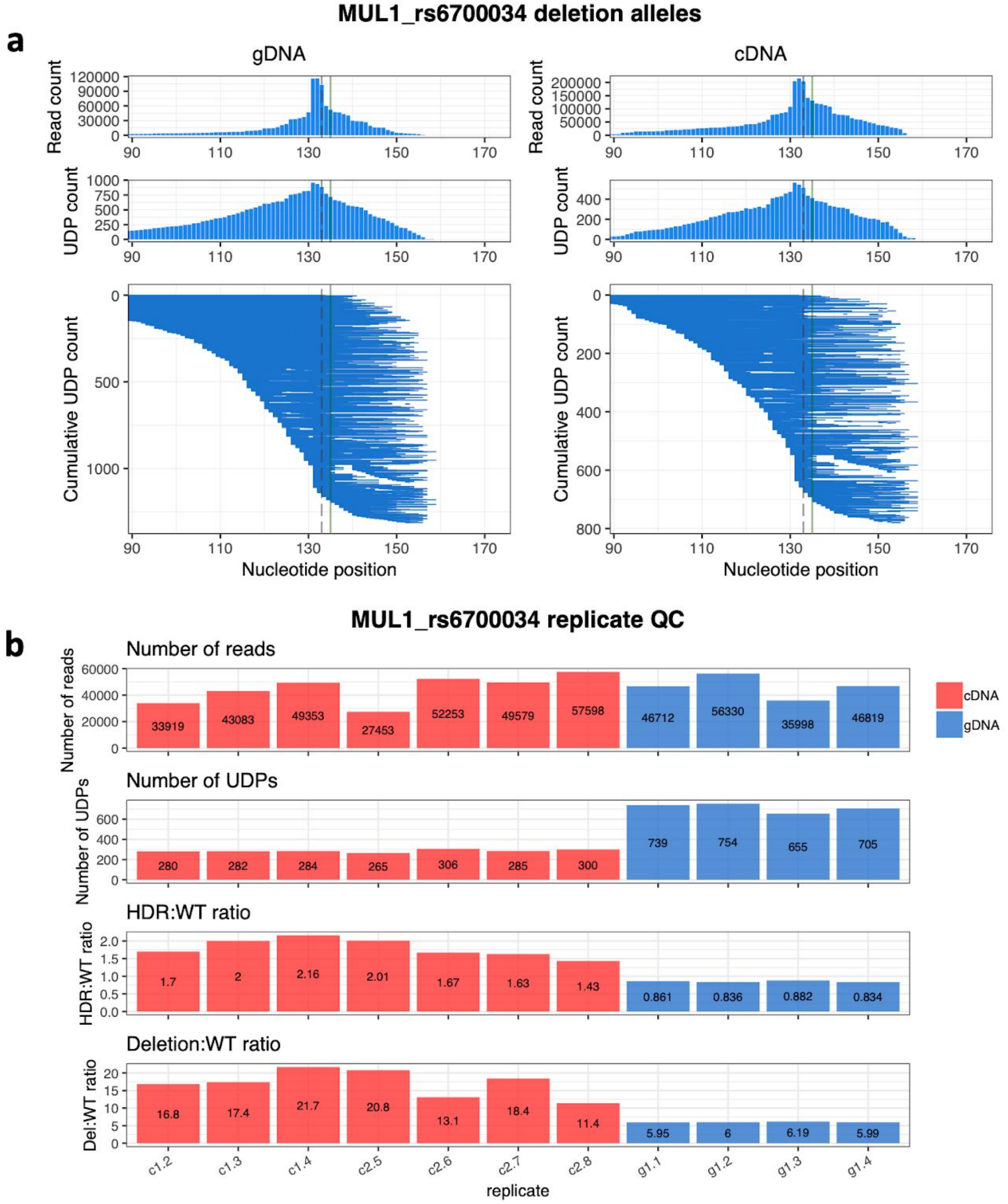

Supplementary Figure 6: Quality control plots for *MUL1*. (a) Deletion alleles. (b) Number of reads, number of UDPs, HDR:WT ratio, and deletion:WT ratio for each replicate. Replicates c1 and c2 are from seperate cDNA synthesis, but all others indicate PCR replicates. Replicate "c1.1" was excluded from statistical analyses for the *MUL1* locus.

Supp Fig 7

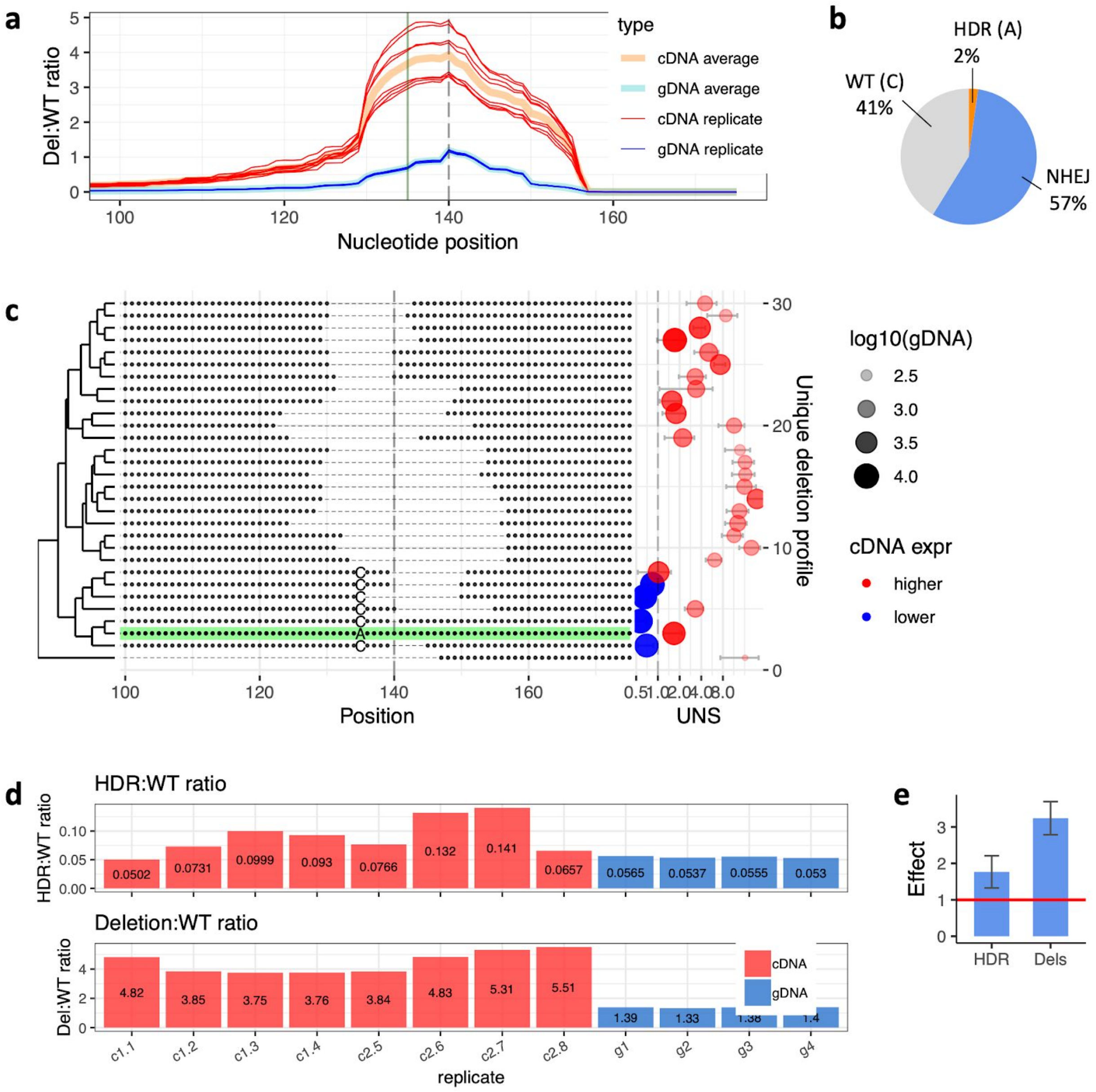

Supplementary Figure 7: Quality control plots for MUL1 targeted with a second guide RNA. (a) Deletion profile plot shows the fraction of reads with a deletion covering each position, divided by the number of WT reads. Deletions over rs6700034 show strong upregulation of expression. (b) Pie chart showing fractions of reads that were wild-type (WT), had deletions (NHEJ), or had the HDR allele. (c) A UNS plot showing the deletion profile of the top 30 alleles by read count, and their estimated expression relative to WT. (d) HDR:WT ratio and deletion:WT ratio for each cDNA and gDNA replicate. (e) Effect size estimates for HDR allele and deletions covering rs6700034, with whiskers showing the 95% confidence interval.

Supp Fig 8

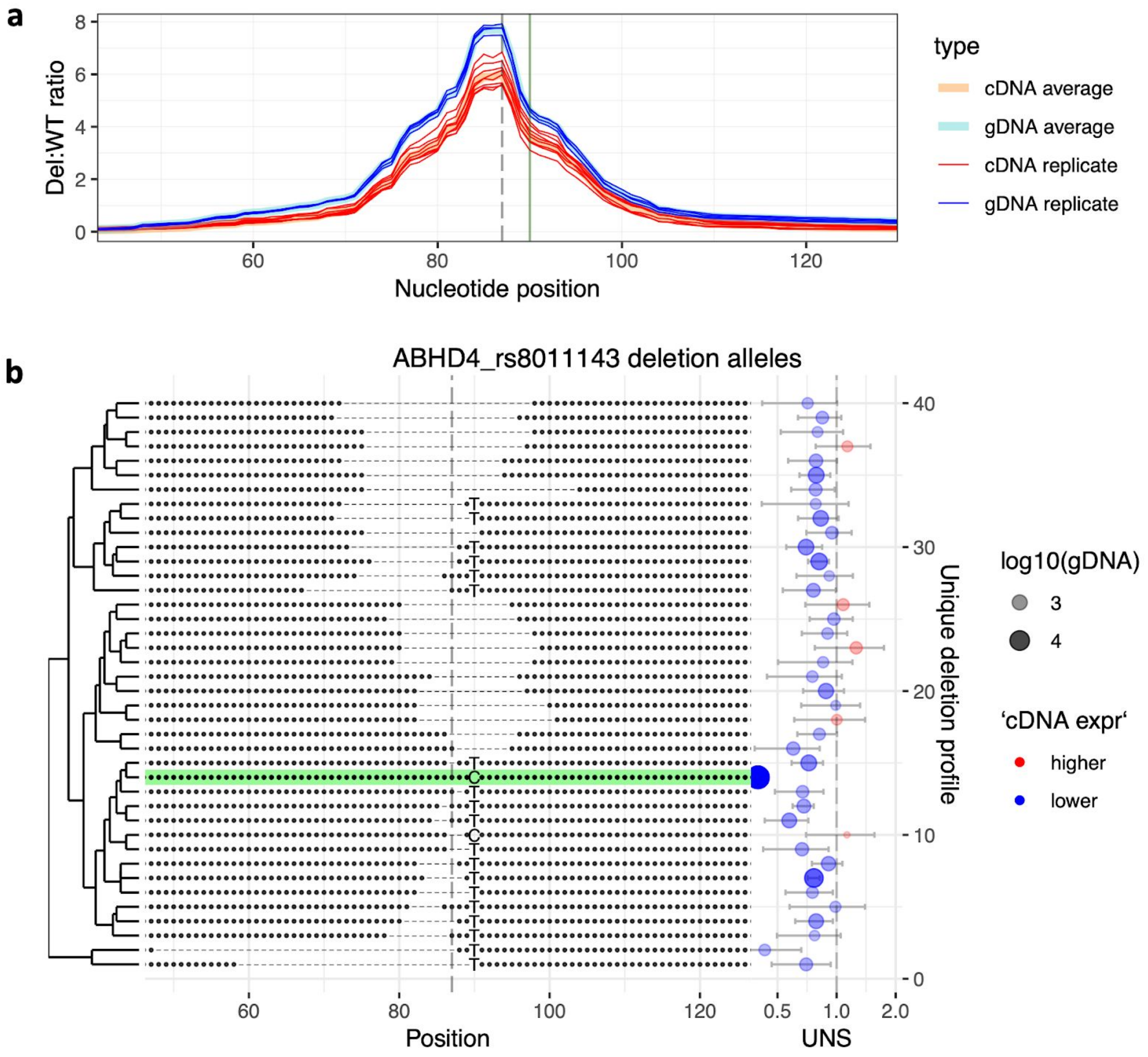

Supplementary Figure 8: *ABHD4* GenIE deletions. **(a)** A deletion profile plot shows, for each position in the *ABHD4* amplicon (80 bp window centred on rs8011143), the fraction of reads with a deletion covering the position, divided by the total number of WT reads. Reads with deletions are downregulated in cDNA relative to their prevalence in gDNA. **(b)** A UDP-normalized score (UNS) plot shows, for the top 40 alleles, the deletion profile of the allele, as well as its estimated expression relative to WT (UNS score). Alleles are clustered according to their deletion profile, and the HDR allele is highlighted green.

Supp Fig 9

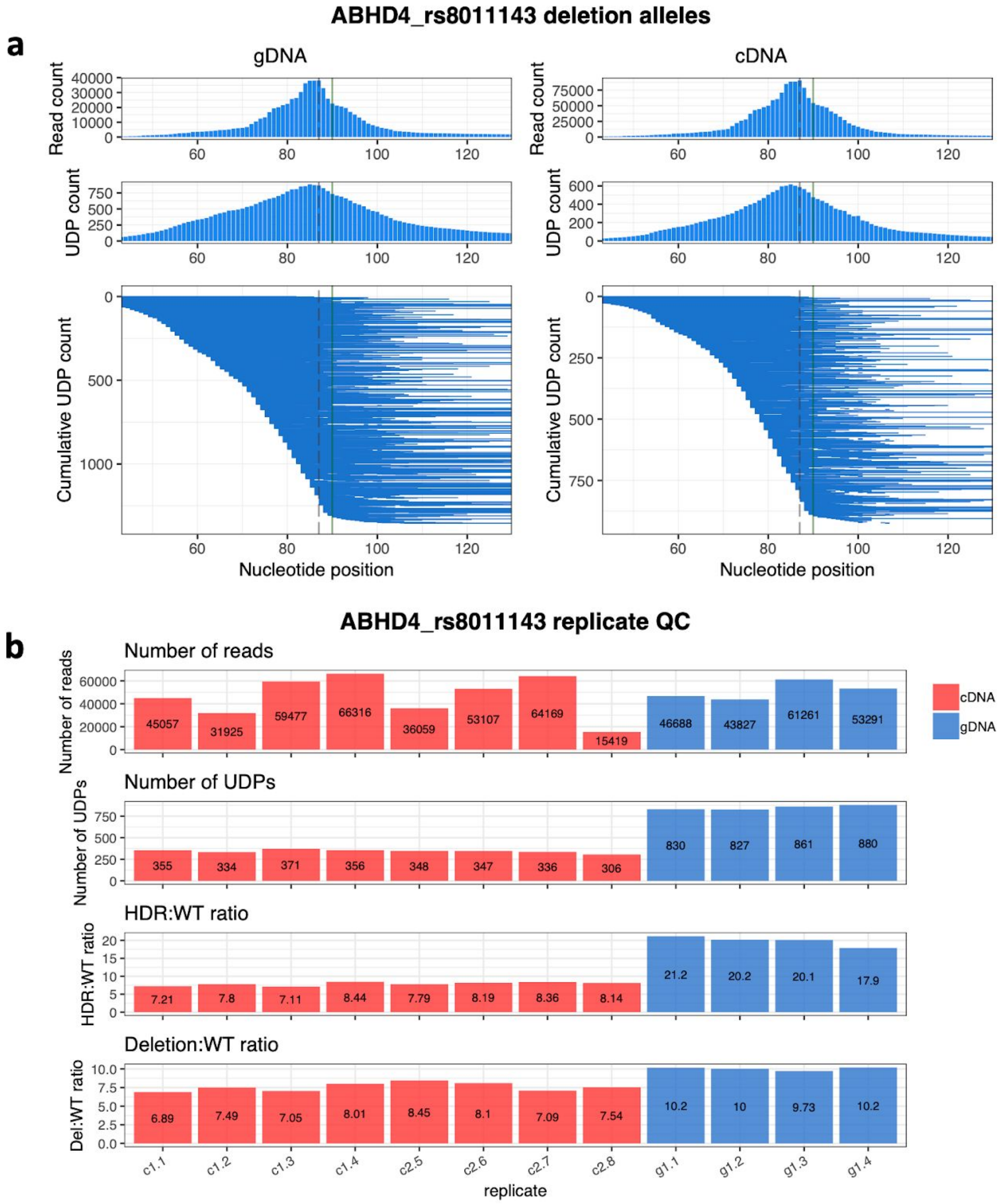

Supplementary Figure 9: Quality control plots for *ABHD4*. (a) Deletion alleles. (b) Number of reads, number of UDPs, HDR:WT ratio, and deletion:WT ratio for each replicate. The HDR introduced allele of rs801143 (C) has lower expression than WT (T).

Supp Fig 10 - *CLU*

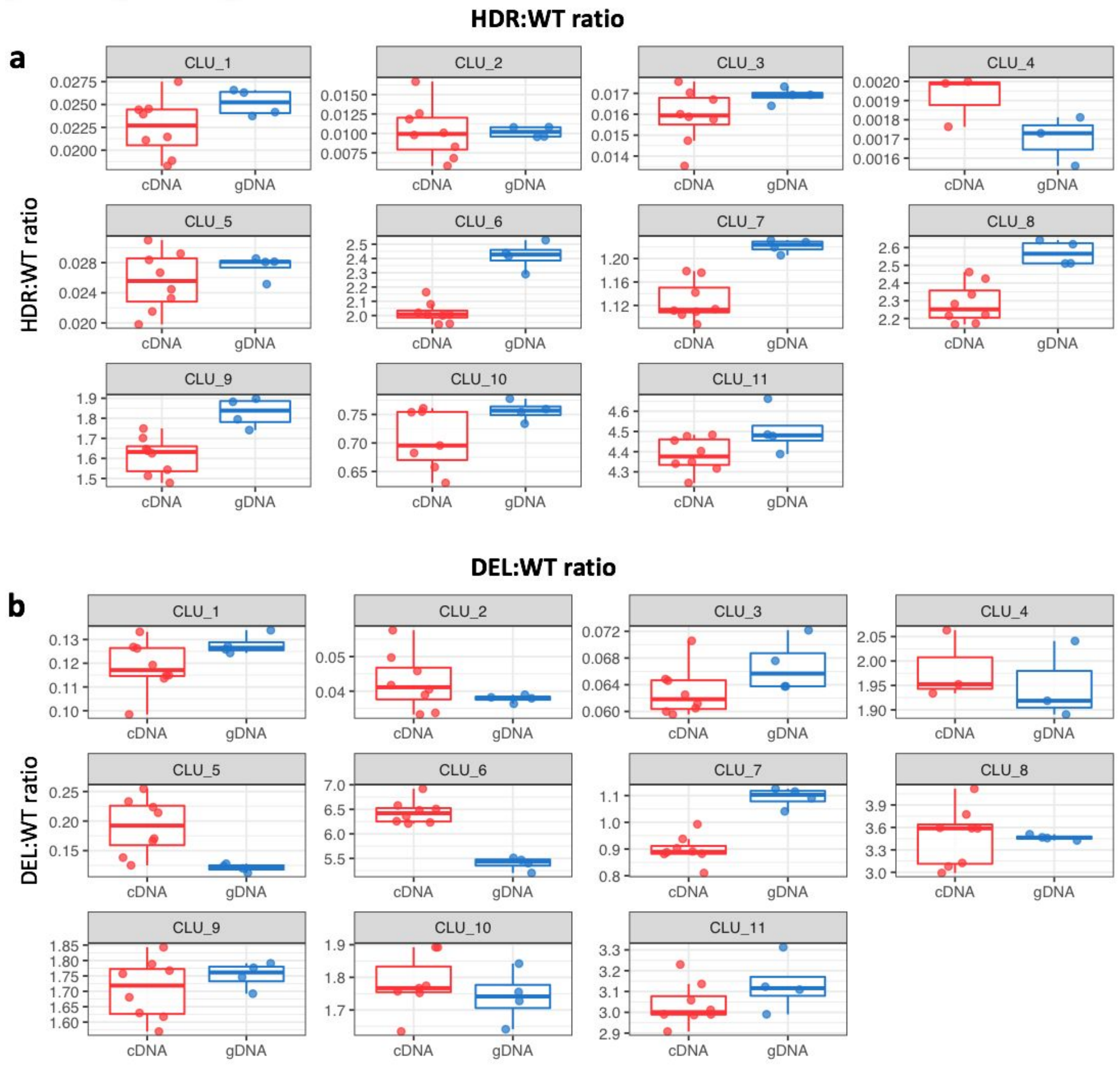

Supplementary Figure 10: Effects for CLU alleles. (a) HDR:WT ratios and (b) deletion:WT ratios for all replicates for each of the 11 CLU alleles targeted. Supplementary Table 2 has details of the HDR and WT allele SNP nucleotides.

Supp Fig 11 - Example of primer positioning for GenIE

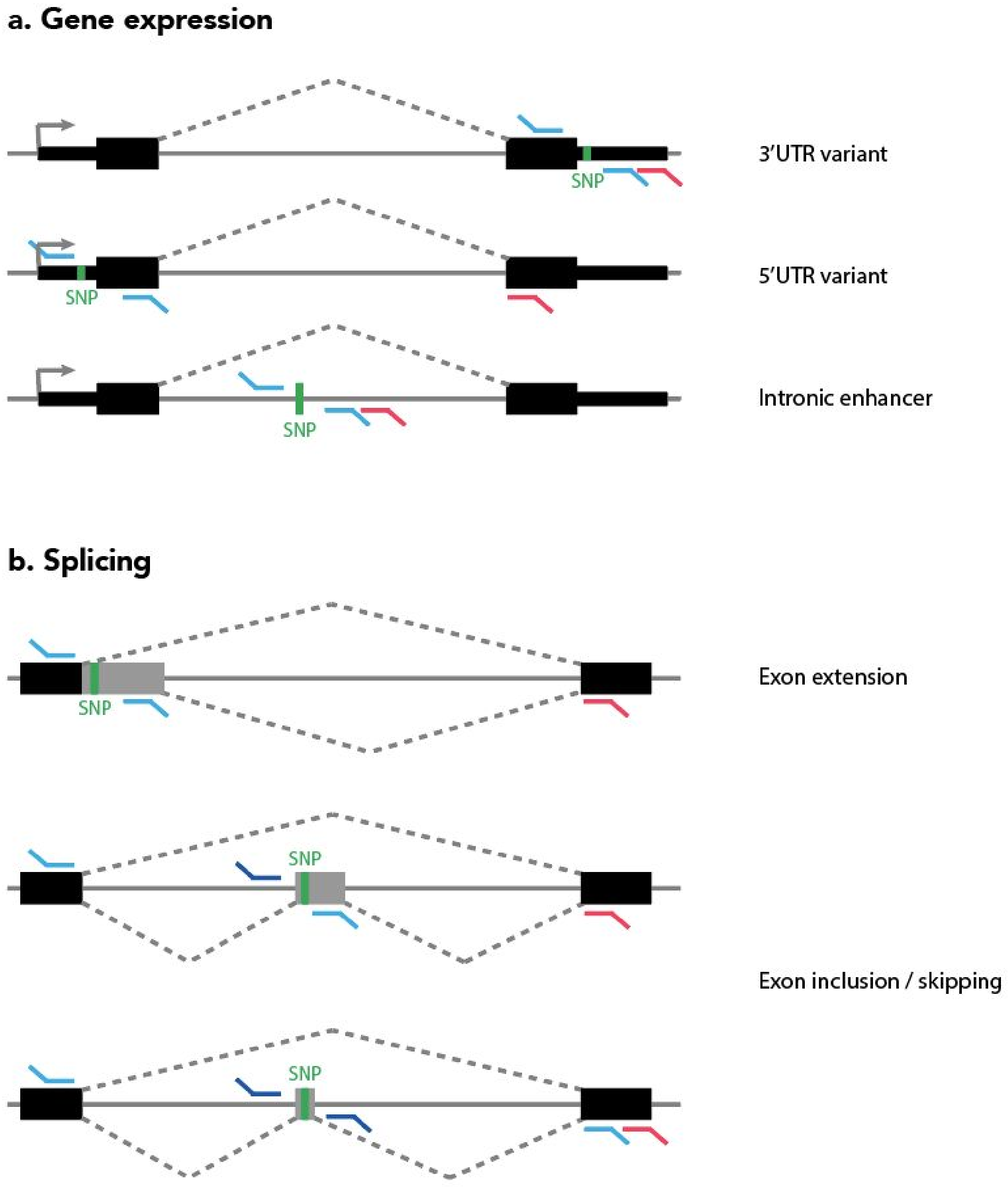

Supplementary Figure 11: Examples of primer positioning for GenIE (a) Gene expression in 3'UTR, 5'UTR or intronic enhancers require primers (blue arrows) to be designed to amplify across the variant of interest (green, SNP) and a reverse transcriptase primer (red arrow). (b) Splicing assays are possible in the cases of exon extension (top) or exon inclusion or skipping (bottom). Grey exons are only present in the alternative spliced isoform. In each case, the variant of interest (green, SNP) and at least one primer (light blue) must be present within an exon in either the normally spliced or alternatively spliced isoform. In the case of exon inclusion or skipping, this will frequently require a seperate primer pair for amplification of gDNA (dark blue arrows), since the intronic sequence would prevent amplification from genomic DNA.

Supp Fig 12

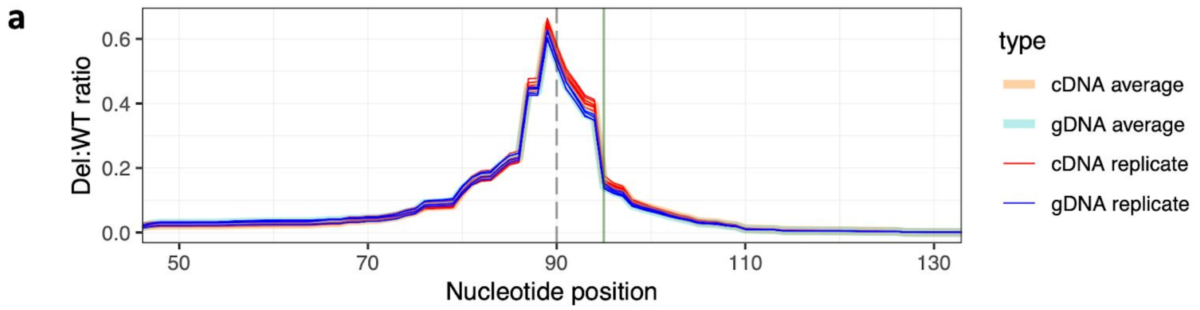

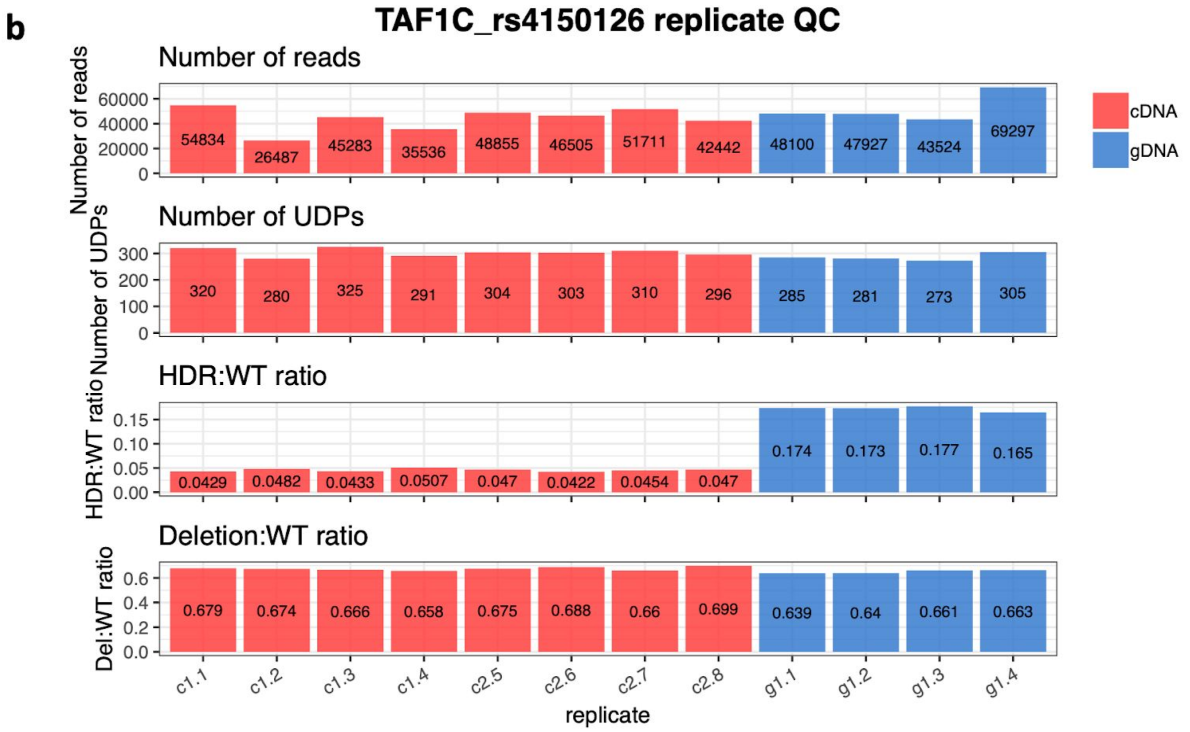

Supplementary Figure 12: Quality control plots for *TAF1C*. (a) Deletion profile plot shows no difference in expression of alleles with deletions over rs4150126. (b) Number of reads, number of UDPs, HDR:WT ratio, and deletion:WT ratio for each replicate. The HDR introduced allele (A) of rs4150126 has lower measured expression than WT (G), which we infer is due to increased usage of the junction 1 splice site.

Supp Fig 13

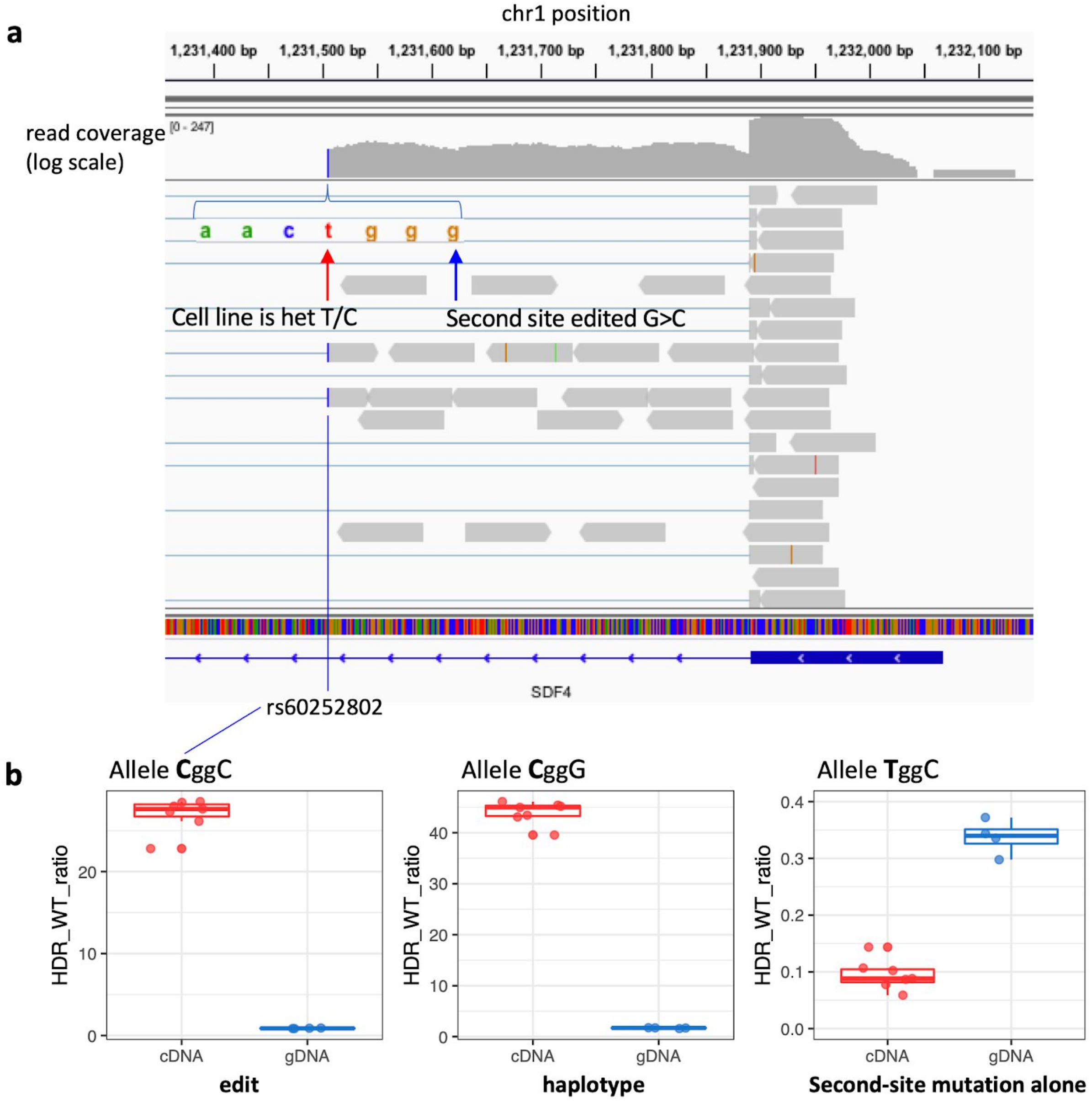

Supplementary Figure 13: *SDF4* splicing. (a) IGV browser view of aligned reads at *SDF4* extended exon in a heterozygous iPSC line. The read coverage (top histogram) is on a log scale. Most reads are spliced at the upstream splice site. However, of the reads which are spliced downstream (near rs60252802), >95% have the C allele at rs60252802. (b) Boxplots showing the HDR:WT ratio of HDR-introduced alleles, relative to the WT allele (TggG). (left) Allele **C**ggC is a mutation of rs60252802 T>C as well as 3 bp distal G>C; (middle) allele **C**ggG is the alternative rs60252802 haplotype; (right) allele **T**ggC is a mutation of only the 3 bp distal G>C, leaving rs60252802 unedited.

Supp Fig 14

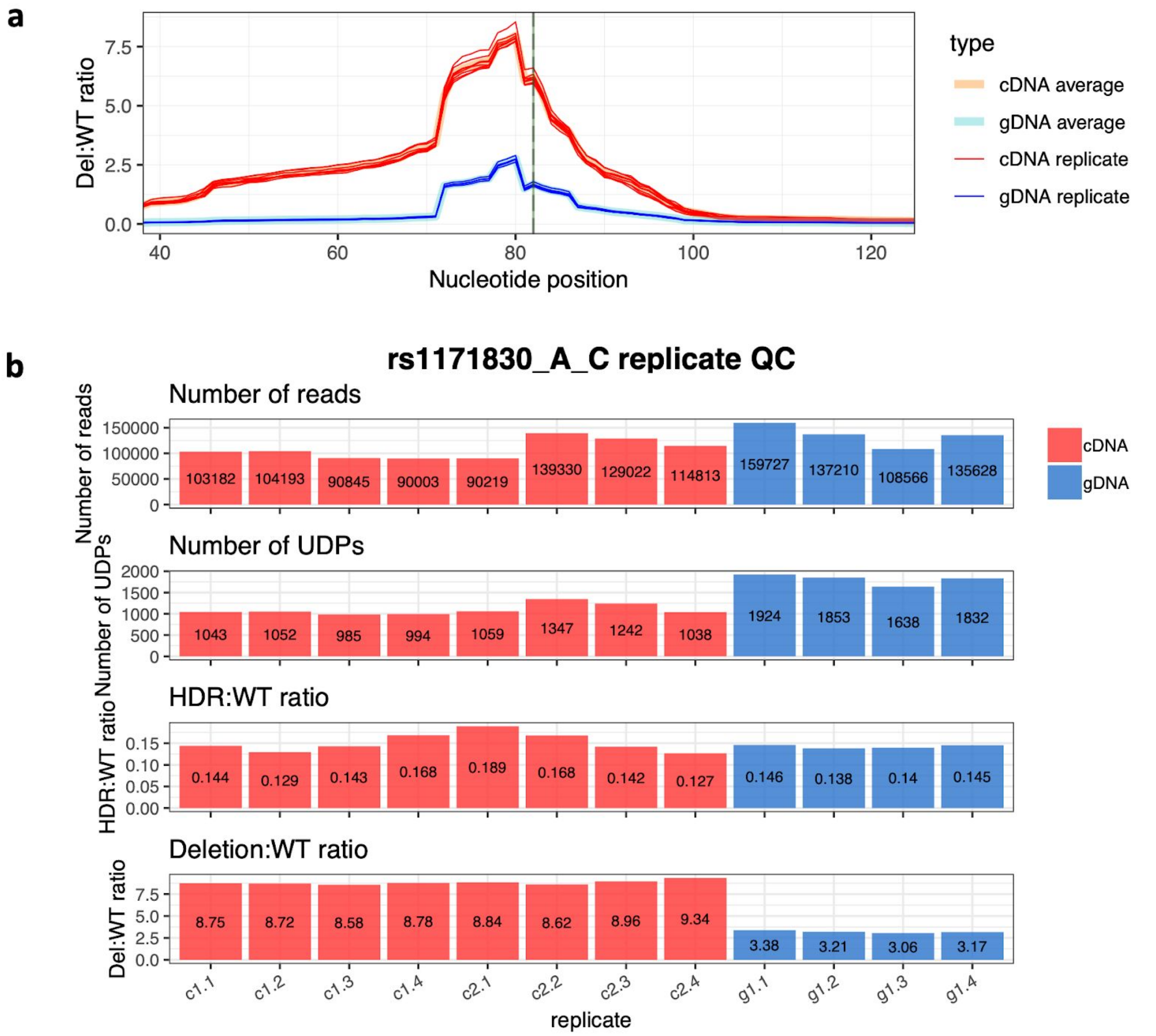

Supplementary Figure 14: Quality control plots for *CCDC6*. (a) Deletion profile plot shows strong upregulation in measured expression (nascent transcript) of alleles with deletions over rsr1171830, due to ablation of the splice site. (b) Number of reads, number of UDPs, HDR:WT ratio, and deletion:WT ratio for each replicate. The HDR introduced allele (C) of rsr1171830 has similar expression to WT (A).

Supplementary References

1. Gaidatzis,D., Burger,L., Florescu,M. and Stadler,M.B. (2015) Analysis of intronic and exonic reads in RNA-seq data characterizes transcriptional and post-transcriptional regulation. *Nat. Biotechnol.*, **33**, 722–729.
